# The Potential for pathogenicity was present in the ancestor of the Ascomycete subphylum Pezizomycotina

**DOI:** 10.1186/1471-2148-10-318

**Published:** 2010-10-21

**Authors:** Aminael Sánchez-Rodríguez, Cindy Martens, Kristof Engelen, Yves Van de Peer, Kathleen Marchal

**Affiliations:** 1 CMPG, Department of Microbial and Molecular Systems, K.U. Leuven, Kasteelpark Arenberg 20, B-3001 Leuven, Belgium; 2 Departments of Plant Systems Biology and Plant Biotechnology and Genetics, Ghent University, Technologiepark 927, B-9052 Ghent, Belgium; 3 Laboratory of Molecular Biology, Institute of Plant Biotechnology, Central University ‘Marta Abreu’ of Las Villas (UCLV), Santa Clara, Cuba

## Abstract

**Background:**

Previous studies in Ascomycetes have shown that the function of gene families of which the size is considerably larger in extant pathogens than in non-pathogens could be related to pathogenicity traits. However, by only comparing gene inventories in extant species, no insights can be gained into the evolutionary process that gave rise to these larger family sizes in pathogens. Moreover, most studies which consider gene families in extant species only tend to explain observed differences in gene family sizes by gains rather than by losses, hereby largely underestimating the impact of gene loss during genome evolution.

**Results:**

In our study we used a selection of recently published genomes of Ascomycetes to analyze how gene family gains, duplications and losses have affected the origin of pathogenic traits. By analyzing the evolutionary history of gene families we found that most gene families with an enlarged size in pathogens were present in an ancestor common to both pathogens and non-pathogens. The majority of these families were selectively maintained in pathogenic lineages, but disappeared in non-pathogens. Non-pathogen-specific losses largely outnumbered pathogen-specific losses.

**Conclusions:**

We conclude that most of the proteins for pathogenicity were already present in the ancestor of the Ascomycete lineages we used in our study. Species that did not develop pathogenicity seemed to have reduced their genetic complexity compared to their ancestors. We further show that expansion of gained or already existing families in a species-specific way is important to fine-tune the specificities of the pathogenic host-fungus interaction.

## Background

The Ascomycetes form the largest phylum in the fungal kingdom. They exhibit a broad range of life styles, ranging from saprophytes to both plant and animal (including human) pathogens. *Mycosphaerella fijiensis*, for instance, is the causal agent of 'Black Sigatoka', one of the most devastating diseases affecting banana and plantains worldwide [1], while the opportunistic pathogen *Aspergillus fumigatus *is the cause of allergy in human patients with atopic immune systems [2]. With the increasing number of genomes of Ascomycetes being available, the study of the evolutionary dynamics that give rise to pathogenicity becomes amenable. The phylogeny of the Ascomycetes and more specifically of the Pezizomycotina is of particular interest in this respect as some of the pathogens are more closely related to their non-pathogenic counterparts than to other pathogens, emphasizing the importance of the environment in acquiring the pathogenic phenotype [3,4].

Recent studies showed that at least for some protein families the retention of duplicated genes is larger in pathogens than in non-pathogens. The functions of such gene families with a larger size in pathogens than in non-pathogens are in line with the pathogenic phenotype. Authors agreed that the presence of pathogen-specific families or the specific increase in gene family size in pathogens created novel gene inventories that can be associated with phenotypic trait acquisitions [3,4]. However, by only comparing gene inventories in the extant species, the evolutionary process that gave rise to the observed enlarged family sizes in pathogens remains unknown. Moreover, most studies which consider gene families at the level of the extant species only tend to explain observed differences in gene family sizes by gains rather than by losses. As a result, the impact of gene loss during genome evolution is usually underestimated.

In this study we evaluated how both gains and losses affect the origin of phenotypic traits that resulted in pathogenicity in Ascomycetes. To this end, we focused on non-pathogenic and plant pathogenic Pezizomycotina. We reconstructed the ancestral states of extant gene families and studied their evolution in relation to their involvement in the phytopathogenic phenotype.

## Results

**Figure 1:**
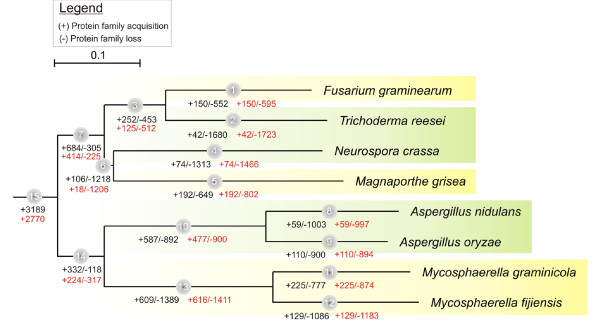
**Evolutionary history in eight Ascomycete species according to DOLLOP and CAFE**. The species tree was derived by NJ (Poisson correction, all nodes 100% BS-supported). Branch lengths are drawn to scale. Branches of the tree corresponding to pathogenic species are shaded in yellow while those corresponding to non-pathogenic species are shaded in green. At each time point (grey circles), the number of gains and losses is indicated as inferred by DOLLOP (black) and CAFE (red). Expansions and contractions of already existing gene families inferred by CAFE are not indicated as these cannot be inferred by DOLLOP.

The phylogenetic relationship between the species used in our analysis is depicted in Figure 1. This tree, derived from a subset of Ascomycete species, shows that not all pathogens cluster together in the species tree. The topology in which some of the pathogens are more closely related to non-pathogenic counterparts is of particular interest to uncover the evolutionary mechanisms that resulted in differential phenotypic trait acquisition (e.g. pathogenicity) and allows to find out which traits are common between evolutionary distant pathogens, whether such common traits have been acquired in each of the pathogens independently (convergent evolution) or whether they have a common origin.

### Gene family size in extant species

**Figure 2:**
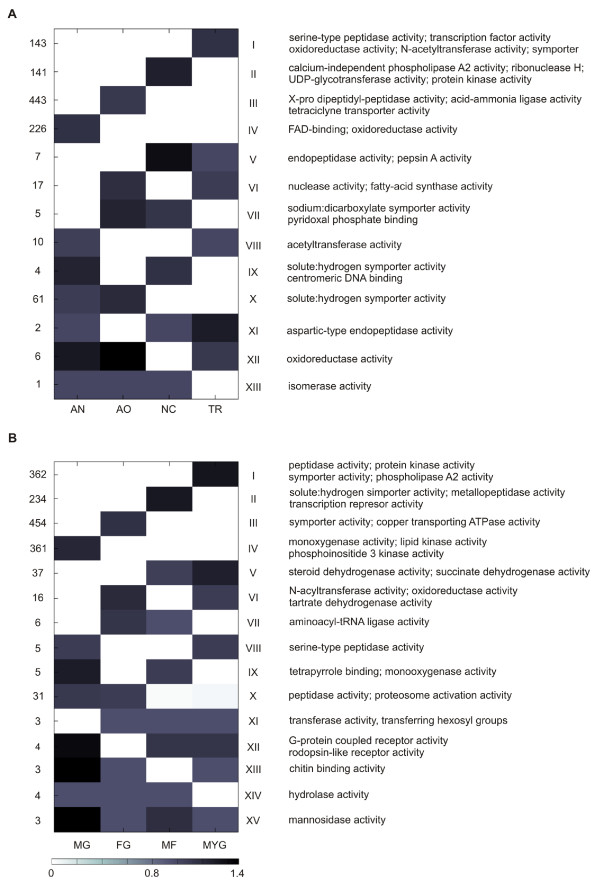
**Clustering of gene families according to their phylogenetic profiles**. Clusters are indicated with roman numbers for A: non-pathogens and B: pathogens. Numbers on the left hand side indicate the number of gene families contained within each cluster (i.e. families with the same normalized phylogenetic profiles). The white-to-black scale, based on the gene family normalized phylogenetic profiles, indicates the size of the families in a cluster per species relative to the family-average (columns). Gene ontology terms of the overrepresented gene families in each cluster are shown at the right. AN: *Aspergillus nidulans*; AO: *Aspergillus oryzae*; NC: *Neurospora crassa*; TR: *Trichoderma reesei*; MG: *Magnaporthe grisea*; FG: *Fusarium graminearum*; MF: *Mycosphaerella fijiensis*; MYG: *Mycosphaerella graminicola*.

For each gene family, a phylogenetic profile was constructed that indicates per species the number of genes belonging to the family. Transforming these profiles allowed extracting those families for which one or more species contained a considerably larger number of genes than observed on average for that family in the remainder of the species; i.e. families that were selectively larger in a certain species or species set (see *Methods*). Subsequent clustering of these families according to the mutual similarity of their normalized profiles identified clusters of families that all displayed a higher than average copy number in the same set of species. Results of this clustering are displayed in Figure 2: some clusters represent families with a copy number that is larger than the family-average in one species only, while other clusters correspond to families that show a copy number that is higher than the average in several species. The latter ones can be further subdivided in gene families with a larger size in species that form a monophyletic clade and those with a larger size in species that do not form a monophyletic clade. Of the 12,530 analyzed gene families, 1066 showed a copy number higher than the average exclusively in non-pathogenic species, while 1528 families had a larger size only in the pathogens. The function of these gene families that showed a copy number that is higher than the family-average in a subset of the species (according to GO and a comprehensive compilation of experimental data (Table 1)) was related to the phenotype of the species in which the larger family size was observed.

**Table 1:** Classification of gene families according to the evolutionary history that explains their origin

Species	Description						Evolutionary events	Class
				
		Clustering	DOLLOP		CAFE			
					
			ancestors	extant	ancestors	extant		
*F. graminearum*	Transmembrane transporter	X	X	X	X	X	gain; TP15; enlarged family size; significant expansion	C1; C3
	Mannosidase activity	X	X		X		TP15; enlarged family size	C3
	Peptidase activity	X		X		X	gain; enlarged family size	C1
	Transferase activity	X		X			gain; enlarged family size	C1
	Chitin binding	X	X		X		TP15; enlarged family size	C3
	Lipid metabolism		X	X	X	X	gain; TP15	C1; C3
	Methyltransferase activity		X		X	X	TP15; significant expansion	C2
	N-acetyl transferase activity	X	X		X		TP15; enlarged family size	C3
	Oxidoreductase activity	X	X		X		TP15; enlarged family size	C3
	Polyssacharide binding			X		X	gain	C1
	Chitinase activity			X		X	gain	C1
	Gluthatione peroxidase			X		X	gain	C1
	Thioredoxin peroxidase			X		X	gain	C1
	Astacin activity		X		X		TP15	C3
	G-coupled protein receptor		X		X		TP15	C3
	Tartrate dehydrogenase activity	X	X		X		TP15; enlarged family size	C3

*T. reesei*	Transmembrane transporters			X		X	loss	C3
	Lipid metabolism			X		X	loss	C3
	Polyketide biosynthetic pathway			X		X	gain	
	Nuclease activity	X	X		X		gain; enlarged family size	
	Chitin binding			X		X	loss	C3
	Astacin activity			X		X	loss	C3
	Fatty-acid synthase activity	X	X		X		gain; enlarged family size	
	N-acetyl transferase activity			X		X	loss	C3
	Mannosidase activity			X		X	loss	C3
	G-coupled protein receptor			X		X	loss	C3
	Oxidoreductase activity			X		X	loss	C3
	Tartrate dehydrogenase activity			X		X	loss	C3

*N. crassa*	Transmembrane transporters			X		X	loss	C3
	Lipid metabolism			X		X	loss	C3
	Chitin binding			X		X	loss	C3
	Astacin activity			X		X	loss	C3
	N-acetyl transferase activity			X		X	loss	C3
	Oxidoreductase activity			X		X	loss	C3
	Mannosidase activity			X		X	loss	C3
	G-coupled protein receptor			X		X	loss	C3
	Tartrate dehydrogenase activity			X		X	loss	C3

*M. grisea*	Mannosidase activity	X	X		X		TP15; enlarged family size	C3
	Peptidase activity	X		X		X	gain; enlarged family size	C1
	G-coupled protein receptor	X	X	X	X		TP15; enlarged family size	C3
	Chitin binding	X	X		X		TP15; enlarged family size	C3
	Lipid metabolism	X	X		X		TP15; enlarged family size	C3
	Transmembrane transporter		X		X		TP15	C3
	Subtilase activity			X		X	gain	C1
	Cutinase activity			X		X	gain	C1
	Polysaccharide binding	X		X		X	gain; enlarged family size	C1
	Chitinase activity	X		X		X	gain; enlarged family size	C1
	Gluthatione peroxidase			X		X	gain	C1
	Thioredoxin peroxidase			X		X	gain	C1
	Ribonuclease H		X		X	X	TP15; significant expansion	C2
	RNA-directed DNA polymerase		X		X	X	TP15; significant expansion	C2
	Astacin activity		X		X		TP15	C3
	Polyketide biosynthetic pathway		X	X	X	X	gain; TP15; significant expansion	C1, C2

*A. nidulans*	Antimicrobial peptide production			X		X	gain	
	Transcription factor activity		X		X	X	gain	
	Light sensing			X		X	gain	
	Chitin binding			X		X	loss	C3
	Astacin activity			X		X	loss	C3
	Lipid metabolism			X		X	loss	C3
	Transmembrane transporter			X		X	loss	C3
	N-acetyl transferase activity			X		X	loss	C3
	Mannosidase activity			X		X	loss	C3
	G-coupled protein receptor			X		X	loss	C3
	Oxidoreductase activity			X		X	loss	C3
	Tartrate dehydrogenase activity			X		X	loss	C3

*A. oryzae*	X- Pro dipeptidyl-peptidase activity	X		X		X	gain; enlarged family size	
	Acid-ammonia ligase activity	X		X		X	gain; enlarged family size	
	Nuclease activity	X	X		X		gain; enlarged family size	
	Fatty-acid synthase activity	X	X		X		gain; enlarged family size	
	Monooxygenase activity			X		X	gain	
	Chitin binding			X		X	loss	C3
	Astacin activity			X		X	loss	C3
	Lipid metabolism			X		X	loss	C3
	Transmembrane transporter			X		X	loss	C3
	Positive regulation of cell growth			X		X	loss	
	N-acetyl transferase activity			X		X	loss	C3
	Mannosidase activity			X		X	loss	C3
	G-coupled protein receptor			X		X	loss	C3
	Oxidoreductase activity			X		X	loss	C3
	Tartrate dehydrogenase activity			X		X	loss	C3

*M.graminicola*	Transmembrane transporters	X	X		X	X	TP15, enlarged family size; significant expansion	C3
	Lipid metabolism		X		X		TP15	C3
	Mannosidase activity	X	X		X		TP15; enlarged family size	C3
	Peptidase activity	X		X		X	gain; enlarged family size	C1
	Transferase activity	X		X		X	gain; enlarged family size	C1
	G-coupled protein receptor	X	X		X		TP15; enlarged family size	C3
	Chitin binding	X	X		X		TP15; enlarged family size	C3
	Astacin activity		X	X	X	X	gain; TP15	C1; C3
	Proteolytic activity			X		X	gain	C1
	Phospholipase A2 activity	X		X		X	gain; enlarged family size	C1
	Oxidoreductase activity	X	X		X		TP15; enlarged family size	C3
	N-acetyl transferase activity	X	X		X		TP15; enlarged family size	C3
	Tartrate dehydrogenase activity	X	X		X		TP15; enlarged family size	C3
	Nitrogen catabolism		X		X		loss	C4
	Response to nitrogen starvation		X		X		loss	C4
	Sensing nitrogen levels		X		X		loss	C4

*M. fijiensis*	Transmembrane transporter	X	X		X		TP15; enlarged family size	C3
	Lipid metabolism		X		X		TP15	C3
	Mannosidase activity	X	X		X		TP15; enlarged family size	C3
	Transferase activity	X		X		X	gain; enlarged family size	C1
	G-coupled protein receptor	X	X		X	X	TP15; enlarged family size	C3
	Metalloexopeptidase activity	X		X		X	gain; enlarged family size	C1
	Proteolytic activity			X		X	gain	C1
	Astacin activity		X	X	X	X	gain; TP15; significant expansion	C1; C3
	Glutathione peroxidase			X		X	gain	C1
	Thioredoxin peroxidase			X		X	gain	C1
	Chitin binding		X		X	X	TP15; significant expansion	C3
	Nitrogen catabolism		X		X		loss	C4
	Sensing nitrogen levels		X		X		loss	C4
	Response to nitrogen starvation		X		X		loss	C4

**Figure 3:**
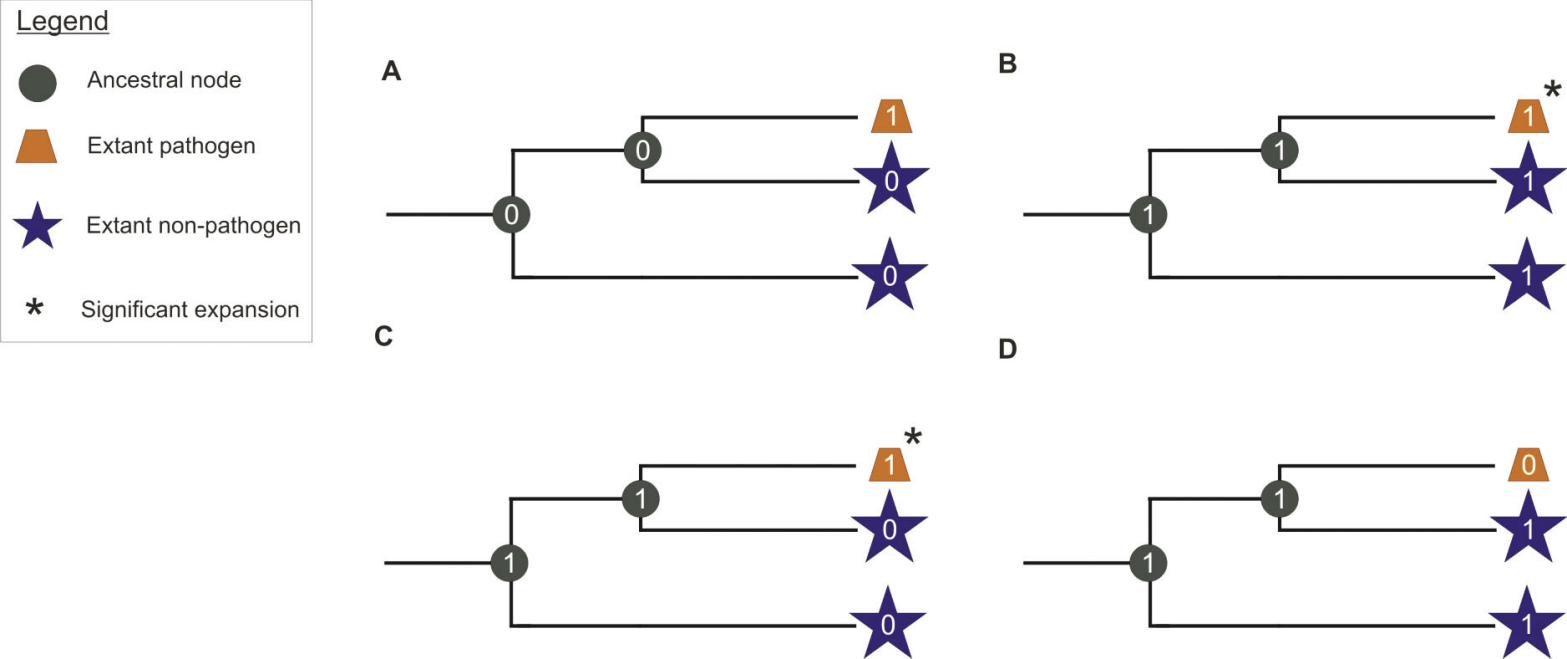
**Evolutionary events explaining gene copy number variations between pathogens and non-pathogens**. Numbers at each node represent the presence ('1') or absence ('0') of members of a hypothetical gene family in each lineage descending from an ancestral species. A) Pathogen-specific gene family gains (Class 1). This event explains the origin of gene families that are not present in an ancestral species common to both pathogenic and non-pathogenic lineages, but appear in one extant pathogen or in an ancestral node that gives rise to exclusively pathogenic lineages. B) Pathogen-specific gene family expansions (Class 2): gene families that are already present in the last common ancestor of the extant species become expanded in a lineage specific way (denoted by a star (*)). C) Non-pathogen-specific losses (Class 3). This event results in gene families that were either present in the last common ancestor of all species or in an ancestor common to both pathogenic and non-pathogenic lineages to become lost selectively in non-pathogenic lineages. Loss of a gene family refers to selective loss of all its members in the considered species or ancestral node. In our study, non-pathogen-specific gene family loss was only observed in extant species. Note that in the pathogenic lineages where members of the considered families were not lost, sometimes a further expansion of the families was observed as denoted by a star (*). D) Pathogen-specific gene family losses (Class 4): gene families either already present in the last common ancestor of all species or in an ancestral species common to both pathogenic and non-pathogenic lineages got lost selectively in pathogenic lineages. In our study, this pathogen-specific gene family loss was also only observed in extant species.

Only families for which the evolutionary history was consistently reconstructed by DOLLOP and CAFE are displayed. **Description**: GO description of the families. **Clustering**: indicates whether an above the family-average gene copy number was observed in at least one species according to the cluster analysis. Evolutionary events in non-pathogens were not categorized. Family size contractions due to gene loss events that do not imply the entire loss of the gene family were not included in the table but covered in the 'Dating gene duplication and loss events' section. **Evolutionary events**: 'gains' and 'losses' as inferred by DOLOP and CAFE; 'significant expansion as inferred by CAFE; 'enlarged gene family size' as inferred by clustering i.e. size of the family in the indicated species is larger than the family average. An 'X' indicates whether the event occurred in an ancestral node or in an extant species. **Class**: Gene families were classified according to the evolutionary event that explains their origin. The four classes (C1-C4) correspond to those shown in Figure 3 and described in the main text.

Remark 1: Non-pathogen-specific gains were never observed.

Remark 2: Although theoretically CAFE should be able to discover significant expansions or contractions of gene families in ancestral species, these were never observed. All cases of significant expansions and contractions were observed in the extant species.

Remark 3: Note that the class distinction was made based on the gene content of the families and not based on their functional annotation. This explains why families belonging to the same GO category can belong to different classes (e.g. some astacin families that were present ancestrally got lost in a non-pathogen-specific way: those belong to C3. Other astacin related families were selectively gained in some pathogens. Those belong to C1.

For most gene families, the sizes are selectively larger than the family-average in one of the sampled species only: this was the case for 953 (89.40%) of the 1066 families that were selectively larger in non-pathogens, and for 1411 of 1528 (92.34%) the families that were selectively larger in pathogens. For the non-pathogens it is mainly in the genome of *A. oryzae *that many families show a higher than family-average gene copy number (443). Those families are enriched in acid-ammonia ligase activity, required for the biosynthesis of essential amino acids and X-Pro dipeptidyl-peptidase activity. The latter activity is in line with the ability of *A. oryzae *to secrete proteolytic enzymes to the environment [5]. For the pathogens, it is mainly *F. graminearum*, *M. fijiensis *and *M. graminicola *that show several families with lineage-specific larger sizes in remarkably similar functions (distinct families, but all related to transmembrane transport (clusters I-III, Figure 2B)).

For gene families that exhibited a higher than average copy number in several species simultaneously, no families were found with a larger size in all four non-pathogens. Clusters XI, XII and XIII in Figure 2A are examples of families (nine in total) with a larger size in three of the four non-pathogens. Among the pathogenic species we found three protein families with a higher than the family-average gene copy number in all four pathogens (cluster XV, Figure 2B). These three protein families are involved in mannosidase activity, which is responsible for oligosaccharide catabolism and N-glycan processing. Where mannosidase activity is not essential for morphogenesis and cellular function in the non-pathogen *A. nidulans*, deleting the activity caused severe defect in conidial formation in the human pathogen *Aspergillus fumigatus *[6].

Clusters X-XIV in Figure 2B consist of 45 families with a larger size in three out of the four pathogens. Functional categories enriched in these protein families included peptidase, G-protein receptor, transferase and chitin binding activities, all of which have been related to pathogenicity in Ascomycetes [7].

We found a larger number of protein families with larger sizes in only two genomes (104 for the non-pathogens, 69 for the pathogens). For the largest subset of those families, gene families exhibited a larger size in two species that form a monophyletic clade: from the 104 families, 64 had a larger size in both related *Aspergillus *species (cluster X of Figure 2A). Also for 37 of the 69 families in pathogenic species, the higher copy number was consistently shared by both closely related *Mycosphaerella *species. Gene families with a larger size in two species that do not belong to a monophyletic clade in the species tree are cluster VI in Figure 2A consisting of 17 protein families enriched in nuclease and fatty-acid synthase activities that show a higher copy number in the genomes of the two non-related non-pathogens *T. reesei *and *A. oryzae *and cluster VI of Figure 2B consisting of 16 protein families enriched in N-acetyltransferase, oxidoreductase and tartrate dehydrogenase activities showing increased copy numbers in the non-related pathogenic genomes of *F. graminearum *and *M. graminicola*.

### Evolution of gene family size

Phylogenetic profiling confirms what was also noted in previous studies, namely that the functionality of protein families with a larger gene copy number in extant pathogens is linked to pathogenicity. The analysis of the phylogenetic profiles, however, does not indicate how these families ended up being extended in the extant pathogens. For instance, the families with a larger copy number in multiple species that do not form a monophyletic clade can either originate through independent duplication events in non-related species (convergent evolution) or by duplications in an ancestral species followed by gene loss in some of the descendants. To understand the evolutionary dynamics of these common and specific traits that can be linked to pathogenicity, we applied a suite of complementary analyses. First, we used DOLLOP [8] which maps for each gene family the evolutionary history of gene gains and losses. By reconciling the binary profiles of gene families with the species tree, DOLLOP infers the minimal gene set for the different ancestral nodes using a parsimony approach which assumes gene loss to be irreversible [9]. DOLLOP thus finds for every internal node in the tree whether the species derived from that node gained or lost a specific protein family (Figure 1). However, as DOLLOP uses binarized phylogenetic profiles that indicate whether a certain species contains a representative of a family, it does not contain information on the size of the protein family in each species.

CAFE [10] on the other hand makes also use of the number of genes in each family to model the evolution of gene families. In doing so, CAFE not only reports on gains and losses, but it also estimates the most likely number of genes for each family at each of the ancestral nodes of the species tree. From these results the occurrence of expansions and contractions of gene families along the time points in the species tree can be derived. CAFE infers an expansion at a given node if the number of genes in a gene family augmented compared to its number in a previous ancestral time point. A gain of a gene family can be considered as a particular case of an expansion for which the gene family was not present in the previous ancestral time point. Similarly, a contraction is a reduction in the number of genes in a gene family compared its number in the previous ancestral time point. Gene family loss is again a particular case of a contraction for which an ancestral gene family completely disappears in a descendant species.

The species tree, with for each node an indication on the global number of gained and lost gene families, as derived from DOLLOP and CAFE, is depicted in Figure 1 (expansions and contractions of already existing protein families identified by CAFE were omitted in the comparison as these events cannot be inferred by DOLLOP). To gain insights into the molecular functions and/or biological processes of the gene families that have been lost or gained or were expanded or contracted at each of the different time points, we analyzed the GO functional categories for which the families were enriched. The complete results of DOLLOP and CAFE, i.e. a list of the evolutionary events for each of the time points (TPs) in the species tree can be found on the supporting information (Additional files 1 and 2). Protein families for which both DOLLOP and CAFE were consistent in inferring the same evolutionary history are displayed in Table 1.

### General observations

In Figure 1, TP15 represents the last common ancestor of the eight species under consideration. Small discrepancies in absolute numbers between DOLLOP and CAFE result from the different underlying assumptions between both methods, but in general both methods predict similar tendencies. Since the origin of the ancestral species on TP7 and TP14, gene families seem to have been continuously lost and this loss does not seem to have been compensated for by gene family gains (both confirmed by DOLLOP and CAFE).

When considering the species derived from TP7 (first group of species in the upper part of the tree), gene loss was significantly higher in the non-pathogens than in the pathogens (TP2 and TP4 versus TP1 and TP5). For the second group of species (descending from TP14), this tendency might be masked by the unusually large genome size that seems to have been maintained in the cultivated species *A. oryzae *[5], resulting in gene loss being at least as prominent in the pathogenic *Mycosphaerella *species than in the non-pathogenic *Aspergillus *species (TP10 versus TP13).

In the following, we give a detailed analysis of the evolutionary events that resulted in the currently observed gene copy numbers in extant pathogens.

### Pathogen-specific gene family gains (Class 1)

With a pathogen-specific gain we refer to a gene family not present in a species that is ancestral to both pathogenic and non-pathogenic lineages (TP15), but that appears in one of the extant pathogens (extant in Table 1) or in an ancestral node that gives exclusively rise to pathogenic lineages (such as for both *Mycosphaerella *species) (Figure 3A). Note that the term 'specific' used in the context of this paper should be interpreted as specific relative to the number of species analyzed in this study.

We found several examples of families that were selectively present in a pathogen or in set of pathogens that originated through this mechanism of family gain and of which the function can be related to pathogenicity.

Examples of families that are gained in one specific extant pathogen are, for instance for *M. grisea*, families involved in subtilase and cutinase activity and families related to the polyketide biosynthesis pathway. All of these gene families have been directly linked to the pathogenic phenotype in *M. grisea *[11,12]. In the pathogen *M. graminicola *we observed a significant gain of families containing genes with peptidase and phospholipase A2 activities. These functions are needed for the organization of infective structures in pathogenic fungi. The expression of genes with phospholipase A2 activity is induced in pathogenic fungi during nutrient starvation, which is assumed to be an environmental trigger for host invasion [13]. Families specifically gained by *M. fijiensis *are enriched in metallo-exopeptidase activity which has been related to the morphological development of infective structures in the fungal pathogen *Ustilago maydis *[14]. In the pathogen *F. graminearum *we found some specific gained gene families related to lipid metabolism and peptidase activity that do not occur in any other species. In *F. graminearum *we also observed the gain of specific gene families associated with transmembrane transporters.

The higher than average family size we observed in multiple extant pathogens that form a monophyletic clade in the species tree, are explained by gains in the common ancestor of these related species, maintained in the extant species (DOLLOP and CAFE). An example is the gain of proteolysis related gene families in the common ancestor TP13 of the closely related *M. fijiensis *and *M. graminicola *species. Especially the gain of a family of zinc endopeptidases with astacin activity is interesting as it has been related to the activation of peptides that act as morphogenetic signal for foot activation in *Hydra *[15].

Of particular interest are the different families, all involved in similar biological processes that were gained independently in multiple species, that are not each other close neighbors in the species tree, as these confer examples of convergent evolution. For instance, *F. graminearum *and *M. grisea *both independently gained gene families that are different from each other, but all related to polyssacharide binding and chitinase activity. Chitinases are closely linked to growth and morphological development in fungi: cell expansion and division, spore germination, hyphal branching and septum formation all depend on enzymes with hydrolytic activities including chitinases [16]. In previous work, a larger than expected size of the chitinase family was already observed for *F. graminearum *[17]. Similarly, the genomes of *F. graminearum*, *M. fijiensis *and *M. grisea *gained different sets of families all related to oxidative stress response, such as families involved in glutathione and thioredoxin peroxidase activities. These gene families that were gained independently from each other in evolution could be involved in a generally conserved defence mechanism of fungi against plant mediated oxidative burst [18]. We also observed different families related to transferase activity that were gained independently in *F. graminearum*, *M. fijiensis *and *M. graminicola*.

### Pathogen-specific gene family expansions (Class 2)

Also gene families with an origin ancestral to both pathogens and non-pathogens that expanded in a pathogen-specific way can explain the larger size of gene families in extant pathogens (Figure 3B). Based on CAFE (see *Methods*), 23 such gene families showed a significant expansion in extant pathogenic species (Additional file 3). For instance, a gene family with methyltransferase activity that has previously been related to fungal growth and virulence [19] showed a significant expansion in *F. graminearum*. As methyltransferases methylate fungal specific glucosylceramides, i.e., molecules that serve as receptors for antifungal plant defensins, they can be directly linked to molecular interactions between the fungus and its host.

Two more gene families with Ribonuclease H and RNA-directed DNA polymerase activities showed a significant expansion in *M. grisea*. Not surprisingly, the function of these families is related to processing transposable elements which are, as indicated by the DOLLOP analysis abundantly present in the genome of *M. grisea*. Insertional mutants of genes from both the Ribonuclease H and RNA-directed DNA polymerase activities gene families are virulence defectives (Table 2). An ancestral family involved in the polyketide biosynthesis pathway was also found to have been expanded in *M. grisea*. The relation of polyketide biosynthesis with pathogenicity in *M. grisea *is strongly supported by the work of Jeon et al. (2007) based on insertional mutagenesis (Table 2).

**Table 2:** Overview of single gene insertional mutants in Ascomycete species

Species	description	Gene ID	Phenotype
*M. grisea*	RNA-dependent DNA replication	MG_12656	reduced virulence; conidiation defective
		MG_11671	reduced virulence; conidiation defective
		MG_13052	reduced virulence; conidiation defective
	Ribonuclease H	MG_13072	reduced virulence; appressorium formation defective
		MG_10039	appressorium formation defective
	Polyketide biosynthetic process	MG_12447	effector (avirulence determinant)
	Transmembrane transporters	MG_13624	Reduced virulence
		MG_00447	Unaffected pathogenicity
*F. graminearum*	Polyketide biosynthetic process	FG_12126	Unaffected pathogenicity
		FG_08208	Unaffected pathogenicity
		FG_10548	Unaffected pathogenicity
		FG_10464	Unaffected pathogenicity
		FG_01790	Unaffected pathogenicity
		FG_08795	Unaffected pathogenicity
		FG_04694	Unaffected pathogenicity
*M. graminicola*	Involved in transmembrane transport	MYG_80109	Reduced virulence

Experimental data was collected from two sources: (1) Pathogen-Host Interaction database (PHI-base) [40] and (2) the work of Jeon et al. [41]. Phenotype: based on mutant deviations in conidiation and appressorium formation compared to untransformed control. Effector: gene product or signaling molecule resulting from its enzymatic activity that triggers defensive mechanisms in the plant host. Hence, effectors are usually known as avirulence determinants.

Although contractions of gene families in ancestral nodes can theoretically be inferred by CAFE, we did not observe them.

### Non-pathogen-specific losses (Class 3)

Both previous mechanisms of pathogen-specific gains and pathogen-specific expansions of ancestral gene families only account for 18.4% of the families with enlarged size in pathogens (according to DOLLOP and CAFE). For a considerable fraction of the families (79.3%), the observed gene family sizes in the extant pathogens seems to originate from an ancestral gene family that got lost in extant non-pathogens, but that was selectively maintained in the pathogens (Figure 3C). With 'loss of a family in a particular species or ancestral node' we refer here to an ancestral gene family that lost all of its members in the species of interest descending from the ancestor. The non-pathogen-specific loss of ancestral gene families is in line with the overall observed higher number of gene losses in non-pathogens than in pathogens (most obvious for species deriving from TP7) for the species we used in this study.

The most striking examples of non-pathogen-specific family losses are those observed for the families involved in transmembrane transport (more specifically secondary active transmembrane transporters, cation:sugar symporters and solute:hydrogen symporters), lipid metabolism and chitin binding. These families, shared by all pathogens, were present in a common ancestor on TP15, but got lost in the non-pathogen lineages (TP2, TP4, TP8 and TP9). This indicates that transmembrane transport, lipid metabolism and chitin binding are essential and generic traits in determining pathogenicity in Ascomycetes.

Related to the evolutionary dynamics of gene families involved in lipid metabolism, it seems that not only these families were lost in the non-pathogenic branch (*T. reesei *lost ancestral gene families involved in lipid metabolism on TP2), but also that the pathogen *F. graminearum *gained some extra families in addition to the ones it inherited from its ancestor on TP3 (DOLLOP and CAFE, Table 1). The fact that both non-pathogen-specific loss and species-specific gains are observed for lipid metabolism further strengthens the importance of this function in pathogenicity. The relation between lipid metabolism and pathogenicity is further confirmed by the fact that disrupting the genes encoding for an extracellular lipase responsible for fatty-acid uptake, significantly reduced virulence in Ascomycete pathogens [20]. In addition, a gene expression study of *F. graminearum *isolated from infected plant tissue showed that genes involved in lipid biosynthesis and degradation were differentially expressed during host colonization [20,21].

Transmembrane transport mechanisms are known to play a key role during the early stages of host invasion by functioning as receptors for signals from the environment that trigger spore germination [21]. A mutant of a gene coding belonging to this family of transmembrane transporters in *M. graminicola *showed a reduced virulence phenotype (Table 2).

On the other hand, chitin binding proteins are known as avirulence receptors that interact with the plant host. Genes encoding chitin binding proteins were differentially expressed in a cDNA library of *M. fijiensis *grown in the presence of banana leaves [22].

Other ancestral gene families present on TP15 that got lost in all studied non-pathogens but were retained in the pathogens are those involved in mannosidase activity, astacin activity and the G-coupled protein receptor.

This model of gene family loss in non-pathogens also explains why we observed members of the families related to N-acetyltransferase, oxidoreductase and tartrate dehydrogenase activities only in the genomes of the pathogens *F. graminearum *and *M. graminicola *that do not form a monophyletic clade in the species tree. The utilization of tartrate has been linked to the induction of pathogenicity related genes in *M. graminicola *[23]. These families originated in the ancestral TP15, and got lost in all extant species other than *F. graminearum *and *M. graminicola *(DOLLOP and CAFE). Both *F. graminearum *and *M. graminicola *share wheat as common host (Additional file 4) and those families only being retained in those two pathogens reinforces the importance of the pathogen-host interaction process in the selection of pathogenic traits.

In addition to the examples discussed so far where gene families have been collectively lost during evolution in all non-pathogens, some losses of gene families were more restricted to the non-pathogens of the evolutionary branch that started either from TP7 or the one that descended from TP14 (these are not discussed in detail here).

Some of the ancestral families that got lost in non-pathogens, but were specifically retained in pathogens underwent further expansions as predicted by CAFE. For those cases it is the combined action of non-pathogen-specific loss and pathogen-specific expansions that explains the observed large family size. For instance the gene families involved in transmembrane transporters showed significant expansions in the pathogens *M. graminicola *and *F. graminearum *(CAFE, Table 1). The same was true for the families involved in chitin binding and astacin activity that were both selectively expanded in *M. Fijiensis*.

### Pathogen-specific gene family losses (Class 4)

In a few cases only, the acquisition of the pathogenic phenotype could be related to the loss of ancestrally present gene families in a pathogen-selective way. A significant number of the families that disappeared in the ancestor of both *Mycospaerella *species (TP13) (Figure 3C) are related to nitrogen catabolic processes, to the regulation of nitrogen utilization and sensing and to cellular responses towards nitrogen starvation. Nitrogen starvation has been related to virulence in Ascomycete plant pathogens by acting as an environmental clue for the expression of disease symptoms in the pathosystem *Magnaporthe grisea - Oryza sativa *[24]. Loss of gene families involved in nitrogen metabolism, regulation and sensing could reduce the ability of the fungus to sense environmental nitrogen levels and mimic a continuous nitrogen starvation condition that might activate the pathogenicity transcriptional program in *M. fijiensis *and *M. graminicola*.

### Dating gene duplication and loss events

**Figure 4:**
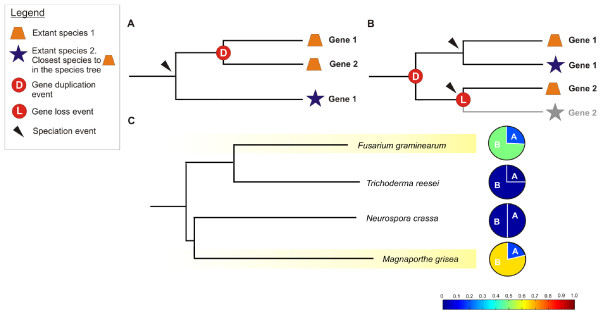
**Origin of the observed variation in gene copy number between Ascomycete species as explained by tree reconciliation**. Panels A and B describe two hypothetical scenarios of how the size of a gene family increased in the extant species: **A**: enlarged gene family size due to a specific expansion in an extant species (species 1). **B**: enlarged gene family size in extant species 1 due to an expansion (duplication) in an ancestral node ('D') before the speciation event between species 1 and 2. Extant species 2 lost one of the copies ('L' in a red circle leading to a gray faded branch) **C**: Tree representing the phylogenetic relations between the studied species. Branches of the tree corresponding to pathogenic species are shaded in yellow. Pie charts indicate, for each of the extant species at the leave of a branch, how many of its gene families with increased size originated through scenario A and B. For instance, for a pathogen, scenario A means that there was a species-specific expansion in the pathogenic branch, while scenario B indicates the gene family in the studied species enlarged because of an ancestral duplication followed by a loss in the non-pathogen, closest to the studied pathogen (see species tree). The color level used in the pie charts (following the blue-to-red scale in below) is an indication for the fraction of enlarged gene families in the studied species, explained by either scenario A or B versus the total number of families included in the phylogenetic analysis. This shows that scenario B occurs much more frequently in absolute numbers in pathogens than in non pathogens.

Both DOLLOP and CAFE agree in predicting that the selective loss of families with an ancestral origin in non-pathogens (i.e. protein families in Class 3) is the major cause for the observed differences in gene copy number between extant pathogenic and non-pathogenic species. So far, predictions made by DOLLOP and CAFE only considered losses of entire gene families (i.e. gene family losses). This is to be expected as DOLLOP can only make predictions about 'presence' or 'absence' (extinct) states of entire protein families while for CAFE the entire loss of a family provide statistically a stronger signal than the spurious loss of a single gene in a family. To uncover whether also less drastic losses i.e., where part of the genes of a family got lost but not the entire family disappears, we relied on tree reconciliation. We analyzed 978 families that have minimally two members in at least one of the species on TP1, TP2, TP4 and TP5 and in addition the family should have representatives in at least two other species (guaranteeing the inference of a reliable tree). We only considered the part of the species tree (Figure 1) where a non-pathogenic species always clusters with its pathogenic counterpart (upper part) as this topology allows directly assessing what proportion of the observed differences in gene family size between extant pathogens and non-pathogens are caused by the different evolutionary scenarios depicted in Figure 4. Gene trees for all families were constructed. By reconciling them with the species tree, we predicted the nodes at which duplication and loss events occurred during evolution of the studies species [25]. To avoid biases introduced by large scale tree reconciliation [26], we retained 445 (45.5%) trees of which the topology was supported with a bootstrap value of at least 75% (the full list of these trees in Newick format can be found in the SI) and only considered the reconciliation results obtained from gene trees with a reliable topology (see materials and methods).

The reconciliation analysis showed that for a relative small fraction of the gene families, larger gene family sizes in the extant pathogens *F. graminearum *and *M. grisea *resulted from expansions of ancestral or later gained gene families specifically occurring in these genomes (Figure 4, B and 4C) (17% of the families in *M. grisea *and 16% of the families in *F. graminearum*). Most expansions occurred selectively in the genome of *M. grisea*. Conversely, expansions of gene families in non-pathogens did barely occur.

The majority of the families with a larger gene copy number in extant pathogens (65% of the families in *M. grisea *and 45.6% of the families in *F. graminearum*) seem to have been the result of ancestral gene families of which the copies were selectively maintained in the pathogens, but got (partially) lost in the non-pathogenic counterparts (Figure 4A and 4C). The observed gene family contractions in the non-pathogens were significantly higher than contractions observed in pathogenic gene families.

## Discussion

Previous comparative approaches studied pathogenicity in Ascomycetes mainly by comparing gene inventories in extant species. In line with these studies we showed that gene families which exhibit a size larger than the family-average in pathogens are responsible for acquired phenotypic traits related to pathogenicity [3,4]. However, by not only studying differences in gene family size between pathogens and non-pathogens, but by also analyzing the complete evolutionary history that gave rise to the observed differences in gene family size, we found that only a relatively small fraction of the gene families with a size that is selectively larger in pathogens could be explained by the combined effect of gene family gain with pathogen-specific expansions.

Most of the families with such larger size in pathogens seemed to have been present already in the ancestor of both pathogens and non-pathogens. The potential of genes and gene products needed to develop pathogenicity was already present in the ancestor of all current species and was selectively maintained in pathogens. Strikingly, it were not the pathogen-specific expansions of these ancestrally present gene families that explained the discrepancies in gene family size between pathogens and non-pathogens, but rather the selective loss of those ancestrally present gene families in non-pathogens. Species that did not develop pathogenicity seem to have reduced their genetic complexity compared to their ancestors. Gene families that evolved according to this gene loss theory are mainly involved in generic functions, conserved amongst all fungi such as transmembrane transport, lipase activity, mannosidase activity, G-coupled protein receptor and astacin activity. Only the number of families with those functions remained considerably higher in pathogens than in non-pathogens. This observation is confirmed by previous molecular studies which showed indeed that a vast repertoire of genes, involved in virulence and pathogenicity, have general functions conserved among all fungal species (e.g. cell surface receptors, members of signaling pathways, transmembrane transporters, members of secondary metabolic pathways) [7].

Pathogenic fungi have to closely interact with their host. It is, therefore, not surprising that the host-pathogen interaction further shaped the genomes of fungal pathogens in the Ascomycetes. We observed that the interaction with a specific environment resulted in the differential retention of gene families with similar functionalities in phylogenetically distant pathogenic species (e.g. families with N-acetyltransferase, oxidoreductase and tartrate dehydrogenase activities shared by the pathogens *F. graminearum *and *M. graminicola*). Moreover, all families that are expanded in certain pathogens could be functionally linked to the specificities of the host-fungus interaction. The latter observations emphasize the importance of the environment in explaining expansions in family size.

## Conclusions

We found that most of the proteins needed to develop pathogenicity were already present in the ancestor of the Ascomycetes lineages we used in our study, i.e. non-pathogenic and plant pathogenic Pezizomycotina. Species that did not develop pathogenicity reduced their genetic complexity compared to their ancestors or pathogenic counterparts. We further showed that expansions of gained or already existing families in a species-specific way are important to fine-tune the specificities of the pathogenic host-fungus interaction.

## Methods

### Species selection

As mentioned before, for the Ascomycetes in general, the pathogenic phenotype does not coincide completely with the species phylogeny. As a result we selected for our analysis 1) a subset of species for which the pathogenic phenotype is in accordance with the species phylogeny (non-pathogens that are each others closest neighbors on the species tree) and 2) a subset of species for which the pathogenic phenotype does not agree with the species phylogeny (pathogen and non-pathogen that are each others closest neighbors in the species tree). Moreover, to avoid biases in our analysis we selected an equal number of non-pathogenic versus pathogenic species. This resulted in the following selection: as non-pathogens we selected *Neurospora crassa *(BROAD Institute, v3.0), *Trichoderma reesei *(DOE Joint Genome Institute, v2.0), *Aspergillus nidulans *(BROAD Institute, v4.0) and *Aspergillus oryzae *(BROAD Institute, v3.0). As pathogens we choose the well studied plant pathogens: *Magnaporthe grisea *(BROAD Institute, v6.0) and *Fusarium graminearum *(BROAD Institute, v3.0) and two economically important plant pathogens, *Mycosphaerella fijiensis *(DOE Joint Genome Institute, v1.0) and *Mycosphaerella graminocola *(DOE Joint Genome Institute, v1.0). As outgroup species we selected the well annotated model organisms: *Saccharomyces cerevisiae *(BROAD Institute, v2.0), *Schizosaccharomyces pombe *(BROAD Institute, v2.0) and *Ustilago maydis *(BROAD Institute, v2.0).

### Construction of gene families

Gene families were reconstructed from the proteomes of the selected species. Pairwise similarities were obtained by an all-against-all BLASTP search using an E-value cutoff of 10E-05. Protein families were derived based on these similarity scores by means of protein clustering. Three different tools for protein clustering, ORTHOMCL [27], BLASTCLUST [28] and GENERAGE [29] were evaluated using a benchmark dataset consisting of experimentally validated gene families collected from the literature (Additional file 5). BLASTCLUST seemed to suffer from transitivity relations that are the result of the multidomain composition of eukaryotic proteins and often created large superfamilies of non-related proteins. Both GENERAGE and ORTHOMCL obtained a high accuracy on the benchmark and performed well with respect to the multidomain composition of proteins. As ORTHOMCL has the advantage of being notably faster than GENERAGE, final gene families were reconstructed using ORTHOMCL (with an inflation factor of 1.5). This resulted in 89,554 individual proteins being clustered based on their sequence similarity into 12,530 families containing two or more members (multiprotein families) and 19,537 singletons i.e., proteins lacking significant homology to other proteins in the dataset.

### Clustering of phylogenetic profiles

For each gene family (12,530 in total), a phylogenetic profile was constructed. Each value in the profile corresponds to the number of genes a particular species contains for that family. To decide, for each family, whether it contains for one or more species a considerably larger number of genes than observed on average for that family in the remainder of the species, we transformed the profiles as follows: 1) every value in a profile equal to '1' was converted to '0' since '1' represents *de novo *gene gains and no enlargement in the corresponding species 2) the profile was then normalized by subtracting its minimum and dividing by its maximum. By this transformation all the values in a profile were rescaled from 0 (meaning no size enlargement at all in a particular species) to 1 (corresponding to the maximum relative size enlargement in a particular species). A gene family was then considered to selectively display a size larger than the family-average in the species where its normalized value exceeded 0.25. Gene families with a profile containing values above 0.25 were clustered according to their normalized phylogenetic profiles. For visualization, the log2-values in the normalized profiles were plotted.

### Functional annotation of proteins and protein families

Proteins and protein families were functionally annotated using Gene Ontology (GO, subcategories molecular function and biological process) [30]. In the first step, all proteins were assigned to GO categories by BLAST2GO [31]. If a protein was assigned to a specific GO level, it was automatically assigned to all parental levels of that GO class. GO annotation at the family level was obtained by listing the GO labels for all the genes that belong to a particular family. A weight, equal to the percentage of genes carrying a particular label was assigned to all the GO labels in the family. Only GO labels with a weight greater than 30% were considered as representative for the family. The statistical significance of functional GO enrichment in protein families groups/clusters was evaluated using the hypergeometric distribution. Multiple hypothesis testing was done by FDR [32].

### Inference of the species tree

One hundred single-copy core protein families (i.e., protein families containing exactly one protein in every studied species) were extracted from the previously obtained phylogenetic profiles of the protein families. For every single-copy core protein family, a multiple alignment was created using ClustalW [33]. Columns in the alignment for which a gap was present in >10% of the sequences were removed. To reduce the chance of including misaligned amino acids, all positions in the alignment left or right from the gap were also removed until a column in the sequence alignment was found for which the residues were conserved in all genes included in our analysis. Conservation was determined as follows: for every pair of residues in the column, the BLOSUM62 value was retrieved. If at least half of the pairs had a BLOSUM62 value > 0, the column was considered conserved. The different multiple alignments were concatenated into one large alignment for which a distance matrix was calculated based on Poisson correction using the software package TREECON [34]. The phylogenetic tree was constructed by neighbor-joining. Bootstrap analysis with 500 replicates was performed to test the significance of the nodes.

### Detection of orthologs

Three fungal species, namely *Saccharomyces cerevisiae*, *Schizossacharomyces pombe *and *Ustilago maydis*, were included as outgroups. Orthologous genes were detected between outgroups and the eight Ascomycete species by the Reciprocal Smallest Distance algorithm (RSD) using default parameters [35].

### Loss and acquisition of gene families

As DOLLOP has already been used successfully in previous studies [36,37], we applied it to our datasets to reconstruct a parsimonious evolutionary history of the studied gene families. The DOLLOP program [8] is based on the Dollo principle of parsimony, which assumes irreversible character loss [9]. In the context of our dataset it means that once a gene family is predicted to be lost in one or more lineages, it can no longer be regained during evolution. As input, a binary matrix derived from the phylogenetic profiles of the protein families was used. In this matrix '0' and '1' indicate the presence or absence of each protein family in each of the eight studied genomes and the 'outgroups'. For the three outgroups, presence or absence of a representative of a protein family was based on the orthology map that was derived as explained in the section "Detection of orthologs".

### Evolution of gene family size

The evolution of gene family size was statistically analyzed by CAFE [10]. CAFE assumes a stochastic birth and death (BD) process to model the changes in gene family size over a phylogeny and to estimate the most likely family size in the ancestral species [38]. The phylogenetic profiles of the gene families and a species tree of which the branch lengths are integer and ultrametric time units (i.e. the sum of the lengths from the root to the extant species should be the same for all paths) were used as input for CAFE. This tree was inferred using the same set of concatenated genes used for constructing the species tree but now inference was done based on the nucleotide sequences. A distance matrix was obtained using the DNADIST tool of the PHYLIP suite [8] from a concatenated multiple alignment of the input sequences. The phylogeny was then inferred by KITSCH, also from the PHYLIP suite which assumes an evolutionary clock model and provides ultrametric trees. The following tree in Newick format was supplied as input: ((((((fgr:15,tre:15):7,(ncr:20,mgr:20):2):11,((myg:16,mfi:16):14,(ani:12,aor:12):18):3):26,sce:59):1,spo:60):2,uma:62). An expectation maximization analysis was performed to estimate the parameter λ which is the probability of both gene gain and loss per unit of time in the phylogeny (for our dataset λ = 0.016 was estimated). CAFE computes a p-value for each of the gene families in the dataset. This p-value indicates how well changes in the number of genes in a family over the phylogeny could be explained by assuming a birth and death model fitted to the whole dataset. CAFE also identifies gene families of which the size in certain species is unlikely under a stochastic BD process. These families are considered to show significant expansions or contractions during evolution.

We used the 'likelihood ratio test' option of CAFE to identify branches along which the size change was larger than expected. This allows identifying the extant species or ancestral nodes where the identified significant expansions or contractions took place.

### Gene tree construction and reconciliation

For each gene family, a multiple sequence alignment was created with ClustalW using default parameters. Alignments were further edited by removing non-informative columns as was done for the species tree (see above). Neighbor-joining trees (with 500 bootstrap replicates) were constructed with PHYLIP [8] using amino acid sequences and simple Poisson-corrected substitution models (λ = 1). Orthologs from the outgroup species were used to root the trees. In cases where no outgroup orthologs existed, unrooted trees were generated. Gene trees were filtered based on bootstrap analysis using a cutoff of 75%. Supported trees were then reconciled with the species tree using Notung [39]. Reconciled trees were further checked to account for possible biases in the phylogenetic tree reconciliation method as reported in Hahn et al [26]. To minimize artefacts of tree reconciliation we only considered trees where after a duplication event at least for one species the paralogs were retained. Tree topologies for which this is not the case often reflect erroneous gene trees and reconciling them with the species tree results in a serious overestimation of the number of loss events.

### Pathogenicity defective mutants in Ascomycete species

Data about experimentally validated pathogenicity factors in Ascomycete genomes were obtained from 1) the Pathogen-Host Interaction database (PHI-base available at http://www.phi-base.org/) [40] which contains an overview of experimental phenotype data subdivided into the following categories: 'reduced virulence', 'loss of pathogenicity', 'unaffected pathogenicity', 'effector'; 2) the study by Jeon et al. [41] which provides a collection of *Magnaporthe grisea *insertional mutants from which they selected 202 new pathogenicity loci based on a phenotype analysis (they recorded mutants with a deviation compared to a non-transformed control in the number of germinated conidia and appressorium formation). Deviating mutants were subdivided according to their observed phenotypes as 'conidiation defective', 'appressorium formation defective' and 'ambiguous'. From those mutants, we selected 64 strains of which the affected locus was located in a coding region.

## Authors' contributions

AS and CM conceived the study. AS, CM and KE performed the analyses. AS, KM and YVdP wrote the manuscript. All authors read and approved the final manuscript.

## Supplementary Material

Additional file 1 **Supplemental Table 1 - Significant gains and losses predicted by DOLLOP at different time points (TP) corresponding to the nodes on the Ascomycetes species tree**. **GO-tree**: M: molecular function, P: biological process. **GO-label**: GO term that was found overrepresented in gene families gained at the respective TP. **P-values**: representing the level of overrepresentation of the corresponding GO term. **Q-values**: False Discovery Rate analysis (only Q-values < 0.05 are reported). **#GF**: number of gene families gained. Click here for file

Additional file 2 **Supplemental Table 2 - Gene family size for extant and ancestral species predicted by CAFE**. **Gene Family**: Number of the gene family. **Tree in Newick format**: The Newick format corresponds to the species tree ((((((FG TR) (NC MG)) ((MYG MF) (AN AO))) SC) SP) UM). FG: *Fusarium graminearum*; TR: *Trichoderma reesei*; NC: *Neurospora crassa*; MG: *Magnaporthe grisea*; MYG: *Mycosphaerella graminicola*; MF: *Mycosphaerella fijiensis*; AN: *Aspergillus nidulans*; AO: *Aspergillus oryzae*; SC: *Saccharomyces cerevisiae*; SP: *Schizosaccharomyces pombe*; UM: *Ustilago maydis*. The numbers indicate the family size per species. **P-value**: CAFE computed value based on a Birth and Death model fitted to the whole dataset. **Gene Family Description**: based on GO labels of the genes contained within the family. Click here for file

Additional file 3 **Supplemental Table 3 - List of the gene families that show a significant expansion according to CAFE**. **Gene Family ID**: ID of the gene family (corresponding to the numbering in Additional file 2). **Description**: based on GO labels of the genes contained within the family. **Tree in Newick format**: The Newick format corresponds to the species tree (((FG TR) (NC MG)) ((MYG MF) (AN AO))). FG: *Fusarium graminearum*; TR: *Trichoderma reesei*; NC: *Neurospora crassa*; MG: *Magnaporthe grisea*; MYG: *Mycosphaerella graminicola*; MF: *Mycosphaerella fijiensis*; AN: *Aspergillus nidulans*; AO: *Aspergillus oryzae*. The numbers indicate the family size per species. The number in bold corresponds to the family size in the species where the largest expansion took place. **Species**: species where the gene family showed an unusual size expansion. **Likelihood ratio**: calculated by CAFE, indicates the likelihood that the change in family size in the mentioned species was greater than expected. Click here for file

Additional file 4 **Supplemental Table 4 - Summary of species attributes**. List of attributes for each of the studied species. Click here for file

Additional file 5 **Supplemental Table 5 - Benchmark dataset consisting of experimentally validated gene families**. **Species**: species of which the gene family composition was experimentally validated. **Family description**: based on the GO labels of the genes contained within the family. **Genes**: members of the gene family. **Source**: reference to the publication from where the data was obtained. Click here for file
