# Supplementary material for: The Potential for pathogenicity was present in the ancestor of the Ascomycete subphylum Pezizomycotina
Source: BMC Evol Biol. 2010 Oct 21;10:318. doi: 10.1186/1471-2148-10-318 (PMC3087541; doi:10.1186/1471-2148-10-318)
Supplement: Additional file 1 — Supplemental Table 1 - Significant gains and losses predicted by DOLLOP at different time points (TP) corresponding to the nodes on the Ascomycetes species tree. GO-tree: M: molecular function, P: biological process. GO-label: GO term that was found overrepresented in gene families gained at the respective TP. P-values: representing the level of overrepresentation of the corresponding GO term. Q-values: False Discovery Rate analysis (only Q-values < 0.05 are reported). #GF: number of gene families gained. [file 1471-2148-10-318-S1.DOC]

**SUPPLEMENTARY INFORMATION**

**Table S1**. Significant gains and losses predicted by DOLLOP at different time points (TP) corresponding to the nodes on the Ascomycetes species tree. GO-tree: M: molecular function, P: biological process. GO-label: GO term that was found overrepresented in gene families gained at the respective TP. P-values: representing the level of overrepresentation of the corresponding GO term; Q-values: False Discovery Rate analysis (only Q-values < 0.05 are reported); #GF: number of gene families gained.

| **GAINS** | | | | | | |
| --- | --- | --- | --- | --- | --- | --- |
| **TP** | **GO-tree** | **GO-label** | **GO-description** | **P-value** | **Q-value** | **#GF** |
| 2 | M | GO:0008236 | serine-type peptidase activity | 4.28e-04 | 0.005457 | 3 |
| 2 | M | GO:0003700 | transcription factor activity | 3.66e-06 | 0.00018666 | 6 |
| 2 | M | GO:0003677 | DNA binding | 3.17e-04 | 0.005389 | 6 |
| 2 | M | GO:0008270 | zinc ion binding | 7.70e-03 | 0.03927 | 5 |
| 2 | M | GO:0004348 | glucosylceramidase activity | 3.62e-03 | 0.02637429 | 1 |
| 2 | M | GO:0004175 | endopeptidase activity | 3.21e-03 | 0.02637429 | 3 |
| 2 | M | GO:0004252 | serine-type endopeptidase activity | 6.92e-05 | 0.0017646 | 3 |
| 2 | M | GO:0017171 | serine hydrolase activity | 7.07e-03 | 0.03927 | 2 |
| 2 | M | GO:0017040 | ceramidase activity | 5.43e-03 | 0.03461625 | 1 |
| 2 | M | GO:0004289 | subtilase activity | 1.43e-03 | 0.014586 | 2 |
| 3 | M | GO:0016407 | acetyltransferase activity | 4.28e-04 | 0.013936 | 6 |
| 3 | M | GO:0046914 | transition metal ion binding | 1.49e-03 | 0.03327667 | 23 |
| 3 | M | GO:0008080 | N-acetyltransferase activity | 1.52e-04 | 0.010184 | 6 |
| 3 | M | GO:0003700 | transcription factor activity | 4.19e-04 | 0.013936 | 13 |
| 3 | M | GO:0008270 | zinc ion binding | 1.51e-04 | 0.010184 | 21 |
| 3 | M | GO:0046872 | metal ion binding | 5.20e-04 | 0.013936 | 27 |
| 3 | P | GO:0043170 | macromolecule metabolic process | 1.64e-12 | 6.1336e-10 | 33 |
| 4 | M | GO:0016773 | phosphotransferase activity | 6.15e-03 | 0.02846667 | 3 |
| 4 | M | GO:0004521 | endoribonuclease activity | 8.44e-04 | 0.00984666 | 2 |
| 4 | M | GO:0004672 | protein kinase activity | 3.67e-03 | 0.02140833 | 3 |
| 4 | M | GO:0032559 | adenyl ribonucleotide binding | 7.32e-03 | 0.02846667 | 4 |
| 4 | M | GO:0005524 | ATP binding | 6.86e-03 | 0.02846667 | 4 |
| 4 | M | GO:0016301 | kinase activity | 1.05e-02 | 0.03340909 | 3 |
| 4 | M | GO:0004540 | ribonuclease activity | 2.11e-03 | 0.0184625 | 2 |
| 4 | M | GO:0016891 | endoribonuclease activity | 2.80e-04 | 0.0049 | 2 |
| 4 | M | GO:0032555 | purine ribonucleotide binding | 1.35e-02 | 0.03634615 | 4 |
| 4 | M | GO:0032553 | ribonucleotide binding | 1.34e-02 | 0.03634615 | 4 |
| 4 | M | GO:0004518 | nuclease activity | 9.18e-03 | 0.03213 | 2 |
| 4 | M | GO:0004519 | endonuclease activity | 3.16e-03 | 0.02140833 | 2 |
| 4 | M | GO:0004523 | ribonuclease H activity | 3.86e-05 | 0.001351 | 2 |
| 4 | P | GO:0006810 | transport | 1.64e-02 | 0.02975 | 3 |
| 4 | P | GO:0044260 | cellular macromolecule metabolic process | 2.10e-02 | 0.02975 | 3 |
| 4 | P | GO:0016310 | Phosphorylation | 8.30e-05 | 0.0006545 | 3 |
| 4 | P | GO:0043412 | biopolymer modification | 8.72e-04 | 0.00211774 | 3 |
| 4 | P | GO:0006796 | phosphate metabolic process | 1.54e-04 | 0.0006545 | 3 |
| 4 | P | GO:0006468 | protein amino acid phosphorylation | 3.04e-05 | 0.0005168 | 3 |
| 4 | P | GO:0019538 | protein metabolic process | 2.06e-02 | 0.02975 | 3 |
| 4 | P | GO:0006464 | protein modification process | 6.78e-04 | 0.001921 | 3 |
| 4 | P | GO:0043687 | post-translational protein modification | 3.08e-04 | 0.0010472 | 3 |
| 4 | P | GO:0044267 | cellular protein metabolic process | 1.75e-02 | 0.02975 | 3 |
| 4 | P | GO:0006793 | phosphorus metabolic process | 1.16e-04 | 0.0006545 | 3 |
| 4 | P | GO:0051234 | establishment of localization | 1.80e-02 | 0.02975 | 3 |
| 6 | M | GO:0004553 | O-glycosidase activity | 2.60e-04 | 0.03146 | 6 |
| 6 | M | GO:0016798 | glycosidase activity | 5.51e-04 | 0.0333355 | 6 |
| 6 | P | GO:0005975 | carbohydrate metabolic process | 1.21e-06 | 0.00028435 | 11 |
| 6 | P | GO:0006343 | establishment of chromatin silencing | 2.48e-05 | 0.002914 | 2 |
| 8 | P | GO:0045314 | eye photoreceptor development | 2.33e-03 | 0.045435 | 1 |
| 8 | P | GO:0002775 | antimicrobial peptide production | 4.65e-03 | 0.0473087 | 1 |
| 8 | P | GO:0002808 | antibacterial peptide biosynthetic process | 4.65e-03 | 0.0473087 | 1 |
| 8 | P | GO:0006139 | nucleic acid metabolic process | 5.76e-04 | 0.033696 | 9 |
| 8 | P | GO:0042478 | eye photoreceptor cell development | 2.33e-03 | 0.045435 | 1 |
| 8 | P | GO:0006955 | immune response | 2.33e-03 | 0.045435 | 2 |
| 8 | P | GO:0002807 | antimicrobial peptide biosynthetic process | 4.65e-03 | 0.0473087 | 1 |
| 8 | P | GO:0019731 | antibacterial humoral response | 4.65e-03 | 0.0473087 | 1 |
| 8 | P | GO:0006952 | defense response | 4.31e-03 | 0.0473087 | 2 |
| 8 | P | GO:0051707 | response to other organism | 3.25e-03 | 0.0473087 | 2 |
| 8 | P | GO:0002780 | antibacterial peptide biosynthetic process | 4.65e-03 | 0.0473087 | 1 |
| 8 | P | GO:0002777 | antimicrobial peptide biosynthetic process | 4.65e-03 | 0.0473087 | 1 |
| 8 | P | GO:0043170 | macromolecule metabolic process | 5.72e-12 | 4.4616e-10 | 11 |
| 8 | P | GO:0002778 | antibacterial peptide production | 4.65e-03 | 0.0473087 | 1 |
| 8 | P | GO:0006965 | biosynthesis of antibacterial peptides | 2.33e-03 | 0.045435 | 1 |
| 8 | P | GO:0050830 | defense response to bacterium | 2.33e-03 | 0.045435 | 1 |
| 8 | P | GO:0002816 | biosynthesis of antibacterial peptides | 2.33e-03 | 0.045435 | 1 |
| 9 | M | GO:0015198 | oligopeptide transporter activity | 1.51e-03 | 0.039425 | 2 |
| 9 | M | GO:0004497 | monooxygenase activity | 2.38e-03 | 0.039425 | 5 |
| 9 | M | GO:0005506 | iron ion binding | 2.49e-03 | 0.039425 | 5 |
| 9 | M | GO:0042936 | dipeptide transporter activity | 2.33e-04 | 0.0110675 | 2 |
| 9 | M | GO:0016491 | oxidoreductase activity | 3.24e-06 | 0.0003078 | 16 |
| 9 | M | GO:0015197 | peptide transporter activity | 1.78e-03 | 0.039425 | 2 |
| 9 | P | GO:0042524 | negative regulation of tyrosine phosphorylation of Stat5 protein | 1.18e-05 | 0.0039648 | 2 |
| 9 | P | GO:0042939 | tripeptide transport | 1.17e-04 | 0.019656 | 2 |
| 10 | M | GO:0003677 | DNA binding | 1.62e-04 | 0.014688 | 53 |
| 10 | M | GO:0046914 | transition metal ion binding | 4.36e-04 | 0.029648 | 67 |
| 10 | M | GO:0008270 | zinc ion binding | 8.29e-04 | 0.0450976 | 53 |
| 10 | M | GO:0046872 | metal ion binding | 3.92e-06 | 0.00106624 | 84 |
| 10 | M | GO:0043167 | ion binding | 2.69e-05 | 0.0036584 | 84 |
| 10 | P | GO:0006139 | nucleobase, nucleoside, nucleotide and nucleic acid metabolic process | 9.78e-08 | 1.3985e-05 | 87 |
| 10 | P | GO:0006351 | transcription, DNA-dependent | 3.72e-06 | 0.00029595 | 43 |
| 10 | P | GO:0060255 | regulation of macromolecule metabolic process | 2.31e-04 | 0.00825825 | 42 |
| 10 | P | GO:0044249 | cellular biosynthetic process | 1.00e-03 | 0.02979167 | 62 |
| 10 | P | GO:0043170 | macromolecule metabolic process | 2.46e-13 | 8.7945e-11 | 122 |
| 10 | P | GO:0050794 | regulation of cellular process | 4.03e-05 | 0.00204013 | 74 |
| 10 | P | GO:0006350 | transcription | 2.25e-13 | 8.7945e-11 | 68 |
| 10 | P | GO:0043283 | biopolymer metabolic process | 8.51e-07 | 8.6923e-05 | 100 |
| 10 | P | GO:0010468 | regulation of gene expression | 1.19e-04 | 0.0044781 | 42 |
| 10 | P | GO:0009608 | response to symbiont | 9.69e-04 | 0.02979167 | 2 |
| 10 | P | GO:0019219 | regulation of nucleobase, nucleoside, nucleotide and nucleic acid metabolic | 1.02e-04 | 0.0040516 | 42 |
| 10 | P | GO:0051252 | regulation of RNA metabolic process | 3.63e-06 | 0.00029529 | 38 |
| 10 | P | GO:0043284 | biopolymer biosynthetic process | 9.40e-10 | 2.2403e-07 | 68 |
| 10 | P | GO:0009058 | biosynthetic process | 5.53e-07 | 6.5899e-05 | 90 |
| 10 | P | GO:0009059 | macromolecule biosynthetic process | 8.72e-05 | 0.0036675 | 70 |
| 10 | P | GO:0006355 | regulation of transcription, DNA-dependent | 7.81e-06 | 0.0004653 | 37 |
| 10 | P | GO:0010467 | gene expression | 7.71e-09 | 1.3781e-06 | 84 |
| 10 | P | GO:0032774 | RNA biosynthetic process | 4.69e-06 | 0.00030485 | 43 |
| 10 | P | GO:0031323 | regulation of cellular metabolic process | 4.92e-04 | 0.01675143 | 43 |
| 10 | P | GO:0045449 | regulation of transcription | 1.96e-05 | 0.001078 | 41 |
| 10 | P | GO:0016070 | RNA metabolic process | 4.13e-06 | 0.0002952 | 60 |
| 10 | P | GO:0019222 | regulation of metabolic process | 4.28e-05 | 0.0020401 | 49 |
| 10 | P | GO:0009610 | response to symbiotic fungus | 9.69e-04 | 0.02979167 | 2 |
| 10 | P | GO:0010556 | regulation of macromolecule biosynthetic process | 7.25e-05 | 0.0032398 | 41 |
| 10 | P | GO:0006139 | nucleobase, nucleoside, nucleotide and nucleic acid metabolic process | 9.78e-08 | 1.398e-05 | 87 |
| 11 | M | GO:0008233 | peptidase activity | 7.38e-08 | 4.819e-06 | 13 |
| 11 | M | GO:0046914 | transition metal ion binding | 2.90e-05 | 0.0003818 | 18 |
| 11 | M | GO:0008270 | zinc ion binding | 1.92e-07 | 5.056e-06 | 18 |
| 11 | M | GO:0046872 | metal ion binding | 1.22e-07 | 4.819e-06 | 22 |
| 11 | M | GO:0004190 | aspartic-type endopeptidase activity | 2.84e-03 | 0.028045 | 3 |
| 11 | M | GO:0008236 | serine-type peptidase activity | 2.86e-05 | 0.0003818 | 6 |
| 11 | M | GO:0043167 | ion binding | 2.98e-07 | 5.8855e-06 | 22 |
| 11 | M | GO:0004194 | pepsin A activity | 5.35e-04 | 0.0060378 | 3 |
| 11 | P | GO:0043170 | macromolecule metabolic process | 5.72e-12 | 8.6944e-11 | 18 |
| 11 | P | GO:0006508 | proteolysis | 3.80e-16 | 7.22e-15 | 13 |
| 12 | P | GO:0043170 | macromolecule metabolic process | 4.53e-12 | 3.0804e-10 | 6 |
| 13 | M | GO:0008233 | peptidase activity | 1.61e-06 | 8.4256e-05 | 23 |
| 13 | M | GO:0008533 | astacin activity | 1.32e-03 | 0.020724 | 2 |
| 13 | M | GO:0046914 | transition metal ion binding | 1.87e-05 | 0.00058718 | 44 |
| 13 | M | GO:0020037 | heme binding | 2.71e-03 | 0.03545583 | 11 |
| 13 | M | GO:0008270 | zinc ion binding | 2.82e-04 | 0.0063248 | 34 |
| 13 | M | GO:0046872 | metal ion binding | 1.08e-03 | 0.01884 | 46 |
| 13 | M | GO:0004190 | aspartic-type endopeptidase activity | 2.13e-03 | 0.03040091 | 5 |
| 13 | M | GO:0016684 | oxidoreductase activity, acting on peroxide as acceptor | 8.62e-07 | 6.7667e-05 | 9 |
| 13 | M | GO:0016491 | oxidoreductase activity | 9.87e-06 | 0.0003873 | 47 |
| 13 | M | GO:0004175 | endopeptidase activity | 6.48e-04 | 0.012717 | 12 |
| 13 | M | GO:0004601 | peroxidase activity | 8.62e-07 | 6.7667e-05 | 9 |
| 13 | M | GO:0043167 | ion binding | 7.70e-05 | 0.0020148 | 50 |
| 13 | P | GO:0016310 | phosphorylation | 2.10e-04 | 0.005565 | 12 |
| 13 | P | GO:0006796 | phosphate metabolic process | 3.38e-04 | 0.00671775 | 13 |
| 13 | P | GO:0006468 | protein amino acid phosphorylation | 2.73e-04 | 0.006201 | 10 |
| 13 | P | GO:0044267 | cellular protein metabolic process | 6.03e-15 | 4.793e-13 | 41 |
| 13 | P | GO:0006793 | phosphorus metabolic process | 2.21e-03 | 0.035139 | 11 |
| 13 | P | GO:0044260 | cellular macromolecule metabolic process | 2.48e-12 | 9.858e-11 | 42 |
| 13 | P | GO:0008643 | carbohydrate transport | 4.64e-04 | 0.0081973 | 7 |
| 13 | P | GO:0019538 | protein metabolic process | 4.45e-14 | 2.3585e-12 | 41 |
| 13 | P | GO:0006508 | proteolysis | 5.52e-11 | 1.7553e-09 | 23 |
| **LOSSES** | | | | | | |
| 1 | M | GO:0004497 | monooxygenase activity | 7.93e-05 | 0.0078771 | 21 |
| 1 | M | GO:0016491 | oxidoreductase activity | 3.93e-07 | 0.0001171 | 86 |
| 1 | M | GO:0043169 | cation binding | 4.78e-05 | 0.0071222 | 76 |
| 1 | M | GO:0043167 | ion binding | 2.20e-04 | 0.01639 | 88 |
| 1 | P | GO:0005975 | carbohydrate metabolic process | 1.59e-05 | 0.008586 | 30 |
| 2 | M | GO:0022891 | substrate-specific transmembrane transporter activity | 2.72e-03 | 0.04828 | 84 |
| 2 | M | GO:0017088 | X-Pro dipeptidyl-peptidase activity | 2.38e-03 | 0.043452 | 3 |
| 2 | M | GO:0050660 | FAD binding | 1.28e-05 | 0.00058422 | 47 |
| 2 | M | GO:0046914 | transition metal ion binding | 5.66e-06 | 0.00033402 | 212 |
| 2 | M | GO:0030246 | carbohydrate binding | 5.75e-06 | 0.00033402 | 23 |
| 2 | M | GO:0030570 | pectate lyase activity | 2.38e-03 | 0.043452 | 3 |
| 2 | M | GO:0015291 | secondary active transmembrane transporter activity | 1.04e-07 | 1.3291e-05 | 53 |
| 2 | M | GO:0016645 | oxidoreductase activity, acting on the CH-NH group of donors | 2.80e-03 | 0.04835676 | 12 |
| 2 | M | GO:0008239 | dipeptidyl-peptidase activity | 1.65e-03 | 0.03195 | 5 |
| 2 | M | GO:0001871 | pattern binding | 2.45e-04 | 0.00652312 | 12 |
| 2 | M | GO:0022804 | active transmembrane transporter activity | 2.35e-04 | 0.00652312 | 69 |
| 2 | M | GO:0048037 | cofactor binding | 5.25e-05 | 0.00186375 | 89 |
| 2 | M | GO:0046872 | metal ion binding | 8.57e-04 | 0.01955796 | 239 |
| 2 | M | GO:0050662 | coenzyme binding | 2.66e-05 | 0.00113316 | 74 |
| 2 | M | GO:0022857 | transmembrane transporter activity | 1.41e-03 | 0.030033 | 99 |
| 2 | M | GO:0004553 | hydrolase activity, hydrolyzing O-glycosyl compounds | 1.41e-08 | 3.0033e-06 | 54 |
| 2 | M | GO:0016837 | carbon-oxygen lyase activity, acting on polysaccharides | 1.41e-03 | 0.030033 | 4 |
| 2 | M | GO:0016491 | oxidoreductase activity | 9.44e-25 | 6.0321e-22 | 281 |
| 2 | M | GO:0005402 | cation:sugar symporter activity | 2.04e-06 | 0.00016294 | 34 |
| 2 | M | GO:0016787 | hydrolase activity | 9.01e-06 | 0.00047978 | 270 |
| 2 | M | GO:0030600 | feruloyl esterase activity | 4.18e-05 | 0.00166938 | 5 |
| 2 | M | GO:0043167 | ion binding | 4.73e-05 | 0.00177792 | 254 |
| 2 | M | GO:0004091 | carboxylesterase activity | 1.20e-04 | 0.00351429 | 18 |
| 2 | M | GO:0004497 | monooxygenase activity | 6.87e-05 | 0.00231048 | 47 |
| 2 | M | GO:0015295 | solute:hydrogen symporter activity | 1.13e-04 | 0.00361035 | 31 |
| 2 | M | GO:0016647 | oxidoreductase activity, acting on the CH-NH group of donors, oxygen acep. | 3.13e-04 | 0.00800028 | 7 |
| 2 | M | GO:0015294 | solute:cation symporter activity | 1.80e-07 | 1.917e-05 | 39 |
| 2 | M | GO:0015144 | carbohydrate transmembrane transporter activity | 1.06e-05 | 0.00052103 | 35 |
| 2 | M | GO:0015233 | pantothenate transporter activity | 1.65e-03 | 0.03195 | 5 |
| 2 | M | GO:0005506 | iron ion binding | 3.72e-04 | 0.0091426 | 45 |
| 2 | M | GO:0015293 | symporter activity | 7.18e-08 | 1.1470e-05 | 40 |
| 2 | M | GO:0016798 | hydrolase activity, acting on glycosyl bonds | 7.18e-09 | 2.2940e-06 | 60 |
| 2 | M | GO:0051119 | sugar transmembrane transporter activity | 3.16e-06 | 0.00022436 | 35 |
| 2 | M | GO:0030247 | polysaccharide binding | 1.51e-04 | 0.00438586 | 14 |
| 2 | M | GO:0030248 | cellulose binding | 5.35e-04 | 0.01266167 | 9 |
| 2 | M | GO:0016806 | dipeptidyl-peptidase and tripeptidyl-peptidase activity | 1.65e-03 | 0.03195 | 5 |
| 2 | M | GO:0005351 | sugar:hydrogen symporter activity | 2.04e-06 | 0.00016294 | 34 |
| 2 | P | GO:0044270 | nitrogen compound catabolic process | 2.11e-04 | 0.027679 | 19 |
| 2 | P | GO:0015849 | organic acid transport | 3.05e-04 | 0.0338 | 20 |
| 2 | P | GO:0045491 | xylan metabolic process | 6.15e-04 | 0.046707 | 5 |
| 2 | P | GO:0006725 | aromatic compound metabolic process | 5.31e-04 | 0.046707 | 36 |
| 2 | P | GO:0000272 | polysaccharide catabolic process | 4.60e-06 | 0.001659 | 17 |
| 2 | P | GO:0016998 | cell wall catabolic process | 5.85e-04 | 0.046707 | 10 |
| 2 | P | GO:0045493 | xylan catabolic process | 4.11e-08 | 1.976e-05 | 10 |
| 2 | P | GO:0045490 | pectin catabolic process | 7.93e-05 | 0.012714 | 5 |
| 2 | P | GO:0009056 | catabolic process | 9.25e-05 | 0.013347 | 87 |
| 2 | P | GO:0045488 | pectin metabolic process | 7.93e-05 | 0.012714 | 5 |
| 2 | P | GO:0044247 | cellular polysaccharide catabolic process | 7.14e-05 | 0.012714 | 14 |
| 2 | P | GO:0015887 | pantothenate transport | 6.15e-04 | 0.046707 | 5 |
| 2 | P | GO:0005975 | carbohydrate metabolic process | 5.25e-12 | 7.55e-09 | 102 |
| 2 | P | GO:0044248 | cellular catabolic process | 6.53e-05 | 0.01271 | 84 |
| 2 | P | GO:0006631 | fatty acid metabolic process | 2.77e-04 | 0.033309 | 23 |
| 2 | P | GO:0009310 | amine catabolic process | 6.54e-05 | 0.012714 | 20 |
| 2 | P | GO:0008643 | carbohydrate transport | 1.87e-08 | 1.3495e-05 | 35 |
| 2 | P | GO:0046942 | carboxylic acid transport | 3.63e-04 | 0.037414 | 20 |
| 2 | P | GO:0051234 | establishment of localization | 6.00e-04 | 0.046707 | 202 |
| 3 | M | GO:0004556 | alpha-amylase activity | 7.10e-06 | 0.0005396 | 4 |
| 3 | M | GO:0016491 | oxidoreductase activity | 7.51e-13 | 2.2834e-10 | 85 |
| 3 | M | GO:0004497 | monooxygenase activity | 2.80e-07 | 4.256e-05 | 23 |
| 3 | M | GO:0016160 | amylase activity | 7.10e-06 | 0.0005396 | 4 |
| 3 | M | GO:0005506 | iron ion binding | 2.04e-05 | 0.00124032 | 20 |
| 3 | P | GO:0044275 | cellular carbohydrate catabolic process | 1.63e-04 | 0.0496335 | 10 |
| 3 | P | GO:0005975 | carbohydrate metabolic process | 6.60e-06 | 0.0040194 | 27 |
| 4 | M | GO:0046914 | transition metal ion binding | 5.75e-10 | 1.472e-07 | 186 |
| 4 | M | GO:0030246 | carbohydrate binding | 9.71e-04 | 0.0210944 | 16 |
| 4 | M | GO:0046906 | tetrapyrrole binding | 7.89e-04 | 0.01923657 | 35 |
| 4 | M | GO:0015291 | secondary active transmembrane transporter activity | 1.05e-06 | 6.72e-05 | 43 |
| 4 | M | GO:0022804 | active transmembrane transporter activity | 8.46e-04 | 0.01968873 | 55 |
| 4 | M | GO:0046872 | metal ion binding | 3.61e-07 | 2.6404e-05 | 207 |
| 4 | M | GO:0004553 | hydrolase activity, hydrolyzing O-glycosyl compounds | 1.34e-06 | 6.8266e-05 | 42 |
| 4 | M | GO:0004181 | metallocarboxypeptidase activity | 1.03e-03 | 0.0210944 | 7 |
| 4 | M | GO:0016491 | oxidoreductase activity | 3.85e-34 | 1.9712e-31 | 248 |
| 4 | M | GO:0005402 | cation:sugar symporter activity | 7.90e-05 | 0.002528 | 26 |
| 4 | M | GO:0016787 | hydrolase activity | 3.46e-04 | 0.00984177 | 210 |
| 4 | M | GO:0043169 | cation binding | 5.81e-09 | 9.9157e-07 | 184 |
| 4 | M | GO:0043167 | ion binding | 2.15e-07 | 1.8346e-05 | 214 |
| 4 | M | GO:0004497 | monooxygenase activity | 2.05e-08 | 2.624e-06 | 48 |
| 4 | M | GO:0015295 | solute:hydrogen symporter activity | 7.54e-06 | 0.00029696 | 29 |
| 4 | M | GO:0015294 | solute:cation symporter activity | 1.33e-06 | 6.8266e-05 | 32 |
| 4 | M | GO:0020037 | heme binding | 2.56e-05 | 0.00093622 | 40 |
| 4 | M | GO:0015144 | carbohydrate transmembrane transporter activity | 5.08e-04 | 0.0130048 | 26 |
| 4 | M | GO:0005506 | iron ion binding | 2.88e-08 | 2.949e-06 | 48 |
| 4 | M | GO:0015293 | symporter activity | 1.60e-06 | 6.8266e-05 | 32 |
| 4 | M | GO:0016798 | hydrolase activity, acting on glycosyl bonds | 1.55e-06 | 6.8266e-05 | 46 |
| 4 | M | GO:0004182 | carboxypeptidase A activity | 4.10e-04 | 0.01104842 | 7 |
| 4 | M | GO:0008237 | metallopeptidase activity | 1.70e-03 | 0.03347692 | 23 |
| 4 | M | GO:0051119 | sugar transmembrane transporter activity | 2.26e-04 | 0.0068065 | 26 |
| 4 | M | GO:0030247 | polysaccharide binding | 1.02e-03 | 0.0210944 | 11 |
| 4 | M | GO:0005351 | sugar:hydrogen symporter activity | 7.90e-05 | 0.002528 | 26 |
| 4 | P | GO:0006810 | transport | 5.28e-09 | 3.028e-06 | 174 |
| 4 | P | GO:0000272 | polysaccharide catabolic process | 1.91e-05 | 0.0031296 | 14 |
| 4 | P | GO:0016998 | cell wall catabolic process | 1.12e-09 | 1.284e-06 | 15 |
| 4 | P | GO:0045493 | xylan catabolic process | 3.26e-04 | 0.04154689 | 6 |
| 4 | P | GO:0005975 | carbohydrate metabolic process | 4.21e-06 | 0.00080481 | 65 |
| 4 | P | GO:0010382 | cell wall metabolic process | 1.45e-08 | 4.1578e-06 | 18 |
| 4 | P | GO:0008643 | carbohydrate transport | 3.87e-06 | 0.00080481 | 26 |
| 4 | P | GO:0051234 | establishment of localization | 1.35e-08 | 4.1578e-06 | 176 |
| 4 | P | GO:0006508 | proteolysis | 1.61e-04 | 0.02308338 | 61 |
| 6 | M | GO:0022891 | substrate-specific transmembrane transporter activity | 1.11e-03 | 0.017057 | 65 |
| 6 | M | GO:0003677 | DNA binding | 1.59e-04 | 0.00318691 | 114 |
| 6 | M | GO:0046914 | transition metal ion binding | 4.93e-12 | 6.8919e-10 | 180 |
| 6 | M | GO:0015291 | secondary active transmembrane transporter activity | 2.90e-03 | 0.04177812 | 31 |
| 6 | M | GO:0016645 | oxidoreductase activity, acting on the CH-NH group of donors | 5.72e-04 | 0.010142 | 11 |
| 6 | M | GO:0022804 | active transmembrane transporter activity | 5.86e-05 | 0.00142182 | 55 |
| 6 | M | GO:0048037 | cofactor binding | 3.45e-04 | 0.00662687 | 65 |
| 6 | M | GO:0046872 | metal ion binding | 5.09e-08 | 3.9108e-06 | 193 |
| 6 | M | GO:0050662 | coenzyme binding | 8.19e-04 | 0.01381410 | 52 |
| 6 | M | GO:0022857 | transmembrane transporter activity | 6.67e-05 | 0.00153743 | 80 |
| 6 | M | GO:0016769 | transferase activity, transferring nitrogenous groups | 1.77e-03 | 0.02632161 | 11 |
| 6 | M | GO:0005275 | amine transmembrane transporter activity | 1.46e-07 | 9.6151e-06 | 24 |
| 6 | M | GO:0030170 | pyridoxal phosphate binding | 3.94e-04 | 0.00726536 | 20 |
| 6 | M | GO:0016684 | oxidoreductase activity, acting on peroxide as acceptor | 8.69e-04 | 0.01381410 | 13 |
| 6 | M | GO:0005342 | organic acid transmembrane transporter activity | 9.94e-07 | 4.5823e-05 | 22 |
| 6 | M | GO:0016491 | oxidoreductase activity | 5.11e-32 | 2.3557e-29 | 227 |
| 6 | M | GO:0005402 | cation:sugar symporter activity | 4.41e-06 | 0.00015542 | 27 |
| 6 | M | GO:0043169 | cation binding | 8.33e-05 | 0.00182863 | 154 |
| 6 | M | GO:0043167 | ion binding | 2.05e-09 | 1.8901e-07 | 203 |
| 6 | M | GO:0015295 | solute:hydrogen symporter activity | 1.05e-05 | 0.0003227 | 27 |
| 6 | M | GO:0004497 | monooxygenase activity | 1.46e-04 | 0.00305936 | 36 |
| 6 | M | GO:0016647 | oxidoreductase activity, acting on the CH-NH group of donors, oxygen acp. | 3.53e-05 | 0.00101708 | 7 |
| 6 | M | GO:0004000 | adenosine deaminase activity | 3.23e-03 | 0.043795 | 3 |
| 6 | M | GO:0015294 | solute:cation symporter activity | 4.32e-05 | 0.00117148 | 27 |
| 6 | M | GO:0015171 | amino acid transmembrane transporter activity | 8.21e-07 | 4.2053e-05 | 20 |
| 6 | M | GO:0003700 | transcription factor activity | 2.56e-15 | 5.9008e-13 | 92 |
| 6 | M | GO:0008270 | zinc ion binding | 5.98e-12 | 6.8919e-10 | 144 |
| 6 | M | GO:0051698 | saccharopine oxidase activity | 3.23e-03 | 0.043795 | 3 |
| 6 | M | GO:0015144 | carbohydrate transmembrane transporter activity | 4.20e-06 | 0.00015542 | 29 |
| 6 | M | GO:0015293 | symporter activity | 5.00e-05 | 0.00128055 | 27 |
| 6 | M | GO:0004601 | peroxidase activity | 8.69e-04 | 0.01381410 | 13 |
| 6 | M | GO:0051119 | sugar transmembrane transporter activity | 4.72e-06 | 0.00015542 | 28 |
| 6 | M | GO:0005351 | sugar:hydrogen symporter activity | 4.41e-06 | 0.00015542 | 27 |
| 6 | M | GO:0046943 | carboxylic acid transmembrane transporter activity | 7.72e-07 | 4.2053e-05 | 22 |
| 6 | P | GO:0006810 | transport | 6.97e-05 | 0.00534947 | 140 |
| 6 | P | GO:0009063 | amino acid catabolic process | 4.57e-04 | 0.01913168 | 13 |
| 6 | P | GO:0044270 | nitrogen compound catabolic process | 5.95e-04 | 0.01957125 | 14 |
| 6 | P | GO:0015849 | organic acid transport | 1.25e-07 | 1.9187e-05 | 21 |
| 6 | P | GO:0006351 | transcription, DNA-dependent | 9.66e-05 | 0.0063549 | 77 |
| 6 | P | GO:0019752 | carboxylic acid metabolic process | 5.69e-05 | 0.00534947 | 59 |
| 6 | P | GO:0046395 | carboxylic acid catabolic process | 5.82e-04 | 0.01957125 | 9 |
| 6 | P | GO:0006865 | amino acid transport | 3.42e-08 | 8.9797e-06 | 20 |
| 6 | P | GO:0051171 | regulation of nitrogen compound metabolic process | 1.83e-04 | 0.00991429 | 10 |
| 6 | P | GO:0006350 | transcription | 2.71e-06 | 0.00031198 | 109 |
| 6 | P | GO:0016054 | organic acid catabolic process | 2.33e-04 | 0.01129437 | 10 |
| 6 | P | GO:0006725 | aromatic compound metabolic process | 4.23e-04 | 0.01855157 | 27 |
| 6 | P | GO:0019219 | regulation of nucleobase, nucleoside, nucleotide and nucleic acid metabolic process | 1.12e-03 | 0.03327484 | 78 |
| 6 | P | GO:0006631 | fatty acid metabolic process | 5.81e-04 | 0.01957125 | 17 |
| 6 | P | GO:0051252 | regulation of RNA metabolic process | 1.70e-06 | 0.00022367 | 72 |
| 6 | P | GO:0043284 | biopolymer biosynthetic process | 1.58e-03 | 0.04547437 | 111 |
| 6 | P | GO:0019740 | nitrogen utilization | 5.82e-04 | 0.01957125 | 9 |
| 6 | P | GO:0009310 | amine catabolic process | 5.95e-04 | 0.01957125 | 14 |
| 6 | P | GO:0008643 | carbohydrate transport | 2.55e-09 | 2.3485e-06 | 29 |
| 6 | P | GO:0006355 | regulation of transcription, DNA-dependent | 8.81e-08 | 1.6228e-05 | 75 |
| 6 | P | GO:0016999 | antibiotic metabolic process | 8.10e-04 | 0.024867 | 6 |
| 6 | P | GO:0006808 | regulation of nitrogen utilization | 1.42e-04 | 0.00817387 | 10 |
| 6 | P | GO:0006559 | L-phenylalanine catabolic process | 8.56e-05 | 0.00606443 | 5 |
| 6 | P | GO:0032787 | monocarboxylic acid metabolic process | 6.65e-04 | 0.02111948 | 24 |
| 6 | P | GO:0015837 | amine transport | 3.90e-08 | 8.9797e-06 | 23 |
| 6 | P | GO:0032774 | RNA biosynthetic process | 1.30e-04 | 0.007982 | 77 |
| 6 | P | GO:0046942 | carboxylic acid transport | 3.08e-08 | 8.9797e-06 | 22 |
| 6 | P | GO:0045449 | regulation of transcription | 6.12e-05 | 0.00534947 | 77 |
| 6 | P | GO:0051234 | establishment of localization | 5.41e-04 | 0.01957125 | 139 |
| 6 | P | GO:0006082 | organic acid metabolic process | 6.42e-05 | 0.00534947 | 59 |
| 6 | P | GO:0006807 | nitrogen compound metabolic process | 3.78e-04 | 0.0174069 | 50 |
| 6 | P | GO:0010556 | regulation of macromolecule biosynthetic process | 2.15e-04 | 0.01100083 | 78 |
| 8 | M | GO:0048037 | cofactor binding | 5.41e-05 | 0.0124971 | 60 |
| 8 | M | GO:0050662 | coenzyme binding | 9.46e-05 | 0.0145684 | 49 |
| 8 | M | GO:0016831 | carboxy-lyase activity | 3.68e-04 | 0.042504 | 11 |
| 8 | M | GO:0016491 | oxidoreductase activity | 1.05e-14 | 4.851e-12 | 171 |
| 8 | P | GO:0006629 | lipid metabolic process | 8.51e-05 | 0.04888995 | 42 |
| 8 | P | GO:0046677 | response to antibiotic | 6.75e-05 | 0.04888995 | 10 |
| 9 | M | GO:0003735 | structural constituent of ribosome | 6.29e-05 | 0.0311875 | 27 |
| 9 | M | GO:0017070 | U6 snRNA binding | 1.25e-04 | 0.0311875 | 4 |
| 9 | P | GO:0040010 | positive regulation of growth rate | 2.25e-04 | 0.02522045 | 20 |
| 9 | P | GO:0006626 | protein targeting to mitochondrion | 8.01e-05 | 0.01649137 | 10 |
| 9 | P | GO:0002119 | nematode larval development | 1.07e-04 | 0.01649137 | 23 |
| 9 | P | GO:0010467 | gene expression | 9.49e-06 | 0.00585058 | 145 |
| 9 | P | GO:0040009 | regulation of growth rate | 9.36e-05 | 0.01649137 | 21 |
| 9 | P | GO:0007007 | inner mitochondrial membrane organization and biogenesis | 1.87e-04 | 0.0240435 | 5 |
| 9 | P | GO:0040008 | regulation of growth | 7.40e-05 | 0.01649137 | 28 |
| 9 | P | GO:0045039 | protein import into mitochondrial inner membrane | 9.87e-05 | 0.01649137 | 5 |
| 9 | P | GO:0006119 | oxidative phosphorylation | 1.95e-04 | 0.0240435 | 12 |
| 9 | P | GO:0043681 | protein import into mitochondrion | 4.43e-05 | 0.01649137 | 9 |
| 9 | P | GO:0045927 | positive regulation of growth | 5.07e-04 | 0.048087 | 23 |
| 9 | P | GO:0007006 | mitochondrial membrane organization and biogenesis | 2.79e-04 | 0.02866725 | 6 |
| 10 | M | GO:0008238 | exopeptidase activity | 6.09e-04 | 0.046893 | 12 |
| 10 | M | GO:0004180 | carboxypeptidase activity | 3.76e-04 | 0.03619 | 8 |
| 10 | M | GO:0016684 | oxidoreductase activity, acting on peroxide as acceptor | 2.99e-05 | 0.0038371 | 12 |
| 10 | M | GO:0016491 | oxidoreductase activity | 1.97e-06 | 0.00075845 | 112 |
| 10 | M | GO:0004601 | peroxidase activity | 2.99e-05 | 0.0038371 | 12 |
| 11 | M | GO:0050662 | coenzyme binding | 3.09e-04 | 0.030591 | 40 |
| 11 | M | GO:0004553 | hydrolase activity, hydrolyzing O-glycosyl compounds | 2.87e-05 | 0.0037884 | 28 |
| 11 | M | GO:0016798 | hydrolase activity, acting on glycosyl bonds | 7.39e-06 | 0.00146322 | 32 |
| 11 | M | GO:0016491 | oxidoreductase activity | 1.02e-16 | 4.0392e-14 | 147 |
| 13 | M | GO:0003677 | DNA binding | 9.09e-14 | 1.2566e-11 | 158 |
| 13 | M | GO:0046914 | transition metal ion binding | 9.28e-12 | 7.3312e-10 | 227 |
| 13 | M | GO:0046872 | metal ion binding | 1.19e-21 | 6.5807e-19 | 251 |
| 13 | M | GO:0016491 | oxidoreductase activity | 2.08e-07 | 1.4378e-05 | 195 |
| 13 | M | GO:0043169 | cation binding | 7.20e-15 | 1.3272e-12 | 206 |
| 13 | M | GO:0043167 | ion binding | 5.23e-12 | 4.8203e-10 | 262 |
| 13 | M | GO:0004497 | monooxygenase activity | 4.47e-05 | 0.00274656 | 41 |
| 13 | M | GO:0003700 | transcription factor activity | 7.26e-13 | 8.0295e-11 | 116 |
| 13 | M | GO:0020037 | heme binding | 5.75e-04 | 0.02890682 | 37 |
| 13 | M | GO:0008270 | zinc ion binding | 1.35e-19 | 3.7327e-17 | 178 |
| 13 | M | GO:0005506 | iron ion binding | 2.72e-04 | 0.0150416 | 39 |
| 13 | P | GO:0006810 | transport | 7.57e-05 | 0.00534895 | 168 |
| 13 | P | GO:0009889 | regulation of biosynthetic process | 3.60e-04 | 0.020085 | 80 |
| 13 | P | GO:0060255 | regulation of macromolecule metabolic process | 4.02e-11 | 4.8934e-09 | 121 |
| 13 | P | GO:0044249 | cellular biosynthetic process | 7.03e-06 | 0.00067236 | 166 |
| 13 | P | GO:0019219 | regulation of nucleobase, nucleoside, nucleotide and nucleic acid metabolic process | 7.97e-12 | 1.084e-09 | 120 |
| 13 | P | GO:0051252 | regulation of RNA metabolic process | 3.44e-18 | 4.606e-15 | 112 |
| 13 | P | GO:0043284 | biopolymer biosynthetic process | 7.87e-11 | 8.7816e-09 | 160 |
| 13 | P | GO:0009058 | biosynthetic process | 1.70e-04 | 0.01034682 | 219 |
| 13 | P | GO:0006355 | regulation of transcription, DNA-dependent | 5.30e-12 | 8.8708e-10 | 114 |
| 13 | P | GO:0006808 | regulation of nitrogen utilization | 3.09e-05 | 0.00275834 | 12 |
| 13 | P | GO:0032774 | RNA biosynthetic process | 2.28e-12 | 4.3613e-10 | 118 |
| 13 | P | GO:0031323 | regulation of cellular metabolic process | 1.42e-09 | 1.4626e-07 | 122 |
| 13 | P | GO:0016070 | RNA metabolic process | 4.16e-05 | 0.0033238 | 139 |
| 13 | P | GO:0019222 | regulation of metabolic process | 8.10e-12 | 1.084e-09 | 139 |
| 13 | P | GO:0006351 | transcription, DNA-dependent | 4.13e-14 | 1.4494e-11 | 118 |
| 13 | P | GO:0051171 | regulation of nitrogen compound metabolic process | 4.22e-05 | 0.0033238 | 12 |
| 13 | P | GO:0006350 | transcription | 3.26e-14 | 1.4494e-11 | 152 |
| 13 | P | GO:0010468 | regulation of gene expression | 5.24e-13 | 1.4032e-10 | 123 |
| 13 | P | GO:0019740 | nitrogen utilization | 1.23e-04 | 0.0078427 | 11 |
| 13 | P | GO:0000270 | peptidoglycan metabolic process | 6.79e-04 | 0.03367337 | 3 |
| 13 | P | GO:0046677 | response to antibiotic | 7.59e-05 | 0.0053489 | 12 |
| 13 | P | GO:0008643 | carbohydrate transport | 2.78e-04 | 0.01618443 | 23 |
| 13 | P | GO:0009059 | macromolecule biosynthetic process | 1.05e-04 | 0.00702975 | 175 |
| 13 | P | GO:0045449 | regulation of transcription | 4.33e-14 | 1.4494e-11 | 117 |
| 13 | P | GO:0051234 | establishment of localization | 4.13e-04 | 0.02212028 | 168 |
| 13 | P | GO:0006807 | nitrogen compound metabolic process | 6.19e-04 | 0.0318785 | 58 |
| 13 | P | GO:0010556 | regulation of macromolecule biosynthetic process | 1.05e-12 | 2.343e-10 | 120 |
