# Supplementary material for: The Potential for pathogenicity was present in the ancestor of the Ascomycete subphylum Pezizomycotina
Source: BMC Evol Biol. 2010 Oct 21;10:318. doi: 10.1186/1471-2148-10-318 (PMC3087541; doi:10.1186/1471-2148-10-318)
Supplement: Additional file 3 — Supplemental Table 3 - List of the gene families that show a significant expansion according to CAFE. Gene Family ID: ID of the gene family (corresponding to the numbering in Additional file 2). Description: based on GO labels of the genes contained within the family. Tree in Newick format: The Newick format corresponds to the species tree (((FG TR) (NC MG)) ((MYG MF) (AN AO))). FG: Fusarium graminearum; TR: Trichoderma reesei; NC: Neurospora crassa; MG: Magnaporthe grisea; MYG: Mycosphaerella graminicola; MF: Mycosphaerella fijiensis; AN: Aspergillus nidulans; AO: Aspergillus oryzae. The numbers indicate the family size per species. The number in bold corresponds to the family size in the species where the largest expansion took place. Species: species where the gene family showed an unusual size expansion. Likelihood ratio: calculated by CAFE, indicates the likelihood that the change in family size in the mentioned species was greater than expected. [file 1471-2148-10-318-S3.DOC]

**SUPPLEMENTARY INFORMATION**

**Table S3. List of the gene families exhibiting a significant accelerated evolution identified by CAFE.**

**Gene Family ID: ID of the gene family (corresponding to the numbering in S2). Description: based on GO labels of the genes contained within the family. Tree in Newick format: The Newick format corresponds to the species tree (((FG TR) (NC MG)) ((MYG MF) (AN AO))). FG: *Fusarium graminearum*; TR: *Trichoderma reesei*; NC: *Neurospora crassa*; MG: *Magnaporthe grisea*; MYG: *Mycosphaerella graminicola*; MF: *Mycosphaerella fijiensis*; AN: *Aspergillus nidulans*; AO: *Aspergillus oryzae*. The numbers indicate the family size per species. The number in bold corresponds to the family size of the species where the accelerated evolution was observed. Species: species where the gene family showed an accelerated evolution. Likelihood ratio: calculated by CAFE, indicates the likelihood that the change in family size in the mentioned species was greater than expected.**

| **Gene**  **Family ID** | **Description** | **Tree in Newick format** | **Species** | **Likelihood**  **ratio** |
| --- | --- | --- | --- | --- |
| 1 | Transposable elements containing family (Ribonuclease H activity) | (((0 1) (0 **260**)) ((3 1) (24 6))) | *M. grisea* | 7.07e71 |
| 2 | No annotated | (((0 0) (0 0)) ((0 **164**) (0 0))) | *M. fijiensis* | 2.90e52 |
| 3 | Transposable elements containing family (RNA-directed DNA polymerase activity) | (((0 0) (0 **103**)) ((0 6) (45 1))) | *M. grisea* | 1.50e27 |
| 4 | Transposable elements containing family (CENP-B protein ) | (((3 0) (1 **108**)) ((1 0) (0 1))) | *M. grisea* | 1.66e29 |
| 5 | Polyketide biosynthetic process | (((9 9) (6 **23**)) ((9 5) (15 16))) | *M. grisea* | 116.33 |
| 6 | Transposable elements containing family (RNA-directed DNA polymerase activity) | (((0 0) (1 **79**)) ((0 0) (0 0))) | *M. grisea* | 6.71e21 |
| 7 | Primary active transmembrane transporter activity | (((**15** 7) (4 5)) ((7 5) (14 17))) | *F. graminearum* | 12.39 |
| 9 | Primary active transmembrane transporter activity | (((9 7) (3 8)) ((**1** 6) (7 12))) | *M. graminicola* | 12.56 |
| 10 | Transposable elements containing family (CENP-B protein ) | (((0 0) (0 **50**)) ((0 0) (0 0))) | *M. grisea* | 6.19e13 |
| 12 | No annotated | (((0 0) (0 0)) ((1 **36**) (0 0))) | *M. fijensis* | 3.71e10 |
| 13 | Transposable elements containing family (Integrase activity ) | (((0 0) (0 **34**)) ((1 0) (0 0))) | *M. grisea* | 1.47e9 |
| 14 | No annotated | (((0 0) (0 0)) ((0 **34)** (0 0))) | *M. fijiensis* | 2.20e10 |
| 15 | No annotated | (((0 0) (0 0)) ((**34** 0) (0 0))) | *M. graminicola* | 2.20e10 |
| 17 | No annotated | (((0 0) (0 0)) ((0 **29**) (0 0))) | *M. fijiensis* | 5.29e8 |
| 20 | No annotated | (((0 0) (0 0)) ((**27** 0) (0 0))) | *M. graminicola* | 1.19e8 |
| 27 | No annotated | (((0 0) (0 0)) ((0 **24**) (0 0))) | *M. fijiensis* | 1.26e7 |
| 32 | No annotated | (((**22** 0) (0 0)) ((0 0) (0 0))) | *F. graminearum* | 3.99e7 |
| 42 | No annotated | (((0 0) (0 0)) ((1 **18**) (0 0))) | *M. fijiensis* | 7.11e4 |
| 48 | Methyltransferase activity | (((**7** 1) (7 0)) ((0 1) (2 0))) | *F. graminearum* | 17.55 |
| 51 | No annotated | (((0 0) (0 **18**)) ((0 0) (0 0))) | *M. grisea* | 3.73e4 |
| 90 | No annotated | (((0 0) (0 0)) ((0 **15**) (0 0))) | *M. fijiensis* | 1.54e4 |
| 91 | No annotated | (((0 0) (0 0)) ((0 **15**) (0 0))) | *M. fijiensis* | 1.54e4 |
| 92 | Transposable elements containing family (Retrotransposon gag protein) | (((0 0) (0 0)) ((**15** 0) (0 0))) | *M. graminicola* | 1.54e4 |
