# Supplementary material for: The Potential for pathogenicity was present in the ancestor of the Ascomycete subphylum Pezizomycotina
Source: BMC Evol Biol. 2010 Oct 21;10:318. doi: 10.1186/1471-2148-10-318 (PMC3087541; doi:10.1186/1471-2148-10-318)
Supplement: Additional file 4 — Supplemental Table 4 - Summary of species attributes. List of attributes for each of the studied species. [file 1471-2148-10-318-S4.DOC]

**SUPPLEMENTARY INFORMATION**

| **Species** | **Genes** | **Classification** | **Life style** | **Host specificity** |
| --- | --- | --- | --- | --- |
| *Aspergillus nidulans* | 10665 | Euascomycete | saprophytic | - |
| *Aspergillus oryzae* | 12063 | Euascomycete | saprophytic | - |
| *Neurospora crassa* | 9822 | Euascomycete | saprophytic | - |
| *Trichoderma reesei* | 9129 | Euascomycete | saprophytic | - |
| *Magnaporthe grisea* | 12832 | Euascomycete | hemibiotrophic | rice, wheat, barley, millet |
| *Fusarium graminearum* | 13321 | Euascomycete | necrotrofic | wheat, barley, corn, potato |
| *Mycosphaerella fijiensis* | 10327 | Euascomycete | hemibiotrophic | banana and plantains |
| *Mycosphaerela graminicola* | 11395 | Euascomycete | hemibiotrophic | wheat |

**Table S4.** Summary of species attributes.
