# Supplementary material for: The Potential for pathogenicity was present in the ancestor of the Ascomycete subphylum Pezizomycotina
Source: BMC Evol Biol. 2010 Oct 21;10:318. doi: 10.1186/1471-2148-10-318 (PMC3087541; doi:10.1186/1471-2148-10-318)
Supplement: Additional file 5 — Supplemental Table 5 - Benchmark dataset consisting of experimentally validated gene families. Species: species of which the gene family composition was experimentally validated. Family description: based on the GO labels of the genes contained within the family. Genes: members of the gene family. Source: reference to the publication from where the data was obtained. [file 1471-2148-10-318-S5.DOC]

**SUPPLEMENTARY INFORMATION**

**Table S5. Benchmark dataset consisting of experimentally validated gene families.**

**Species:** species of which the gene family composition was experimentally validated. **Family description:** based on the GO labels of the genes contained within the family. **Genes:** members of the gene family. **Source:** reference to the publication from where the data was obtained.

| **Species** | **Family description** | **Genes** | **Source** |
| --- | --- | --- | --- |
| *N. crassa* | Lignin oxidase | AN_6830, AN_9170, AN_7389, AN_0878, AN_0901, AN_5397, AN_8581 | Levaseur, A. et al. 2008. Fungal Genetics and Biology 45(5): 638-645 |
| *T. reesei* | Lignin oxidase | TR_122948, TR_54239, TR_124079, TR_102820, TR_121098 | Levaseur, A. et al. 2008. Fungal Genetics and Biology 45(5): 638-645 |
| *M. grisea* | NADPH oxidase | MG_00750, MG_06559, MG_08299 | Kawahara, T. et al. 2007. BMC Evolutionary Biology 7(1): 109-129 |
| *A. nidulans* | NADPH oxidase | AN_5457 | Kawahara, T. et al. 2007. BMC Evolutionary Biology 7(1): 109-129 |
| *F. graminearum* | NADPH oxidase | FG_00739, FG_10807, FG_11195 | Kawahara, T. et al. 2007. BMC Evolutionary Biology 7(1): 109-129 |
| *A. nidulans* | Chitinase | AN_0549, AN_5077, AN_8481, AN_9390 | De Groot, P. et al. 2009. Fungal Genetics and Biology 46(1): S72-S81 |
| *N. crassa* | Glucosylceramide Synthase | NC_01116, NC_01111 | Leipelt, M. et al. 2001. Journal of Biological Chemistry 276(36): 33621-33629 |
| *M. grisea* | Glucosylceramide Synthase | MG_10668 | Leipelt, M. et al. 2001. Journal of Biological Chemistry 276(36): 33621-33629 |
